# Post-COVID-19 condition at 6 months and COVID-19 vaccination in non-hospitalised children and young people

**DOI:** 10.1136/archdischild-2022-324656

**Published:** 2023-01-04

**Authors:** Snehal M Pinto Pereira, Manjula D Nugawela, Natalia K Rojas, Roz Shafran, Kelsey McOwat, Ruth Simmons, Tamsin Ford, Isobel Heyman, Shamez N Ladhani, Emily Y Cheung, Lana Fox-Smith, Emma Dalrymple, Terence Stephenson

**Affiliations:** 1 UCL Great Ormond Street Institute of Child Health, London, UK; 2 Immunisation Department, Public Health England, London, UK; 3 Department of Psychiatry, University of Cambridge, Cambridge, UK; 4 Paediatric Infectious Diseases Research Group, St George's University, London, UK

**Keywords:** adolescent health, child health, COVID-19, paediatrics

## Abstract

**Objectives:**

To describe the physical and mental health of children and young people (CYP) 6 months after infection with SARS-CoV-2 and explore whether this varies by COVID-19 vaccination.

**Design:**

A non-hospitalised, national cohort of people aged 11–17 years old with PCR-confirmed SARS-CoV-2 infection and PCR negatives matched at study invitation, by age, sex, region and date of testing who completed questionnaires 6 months after PCR testing. The questionnaire included 21 symptoms and standardised scales (eg, EQ-5D-Y and Chalder Fatigue Scale).

**Results:**

6407 test-positive and 6542 test-negative CYP completed the 6-month questionnaire: 60.9% of test-positive vs 43.2% of test-negative CYP reported at least one symptom 6 months post-test; 27.6% of test-positive vs 15.9% of test-negative CYP reported 3+ symptoms. Common symptoms at 6 months were tiredness and shortness of breath among both test-positive and test-negative CYP; however, the prevalence of both was higher in test-positive (38.4% and 22.8%, respectively) compared with test-negative CYP (26.7% and 10.9%, respectively). 24.5% test-positive vs 17.8% test-negative CYP met the Delphi research definition of long COVID. Mental health, well-being, fatigue and health-related quality of life scores were similar among test-positive and test-negative CYP 6 months post-test. Similarly, symptomatology was similar among COVID-19-vaccinated and COVID-19-unvaccinated test-positive and test-negative CYP.

**Conclusions:**

Six-months post-PCR testing, CYP who tested positive for SARS-CoV-2 had similar symptoms to those who tested negative, but test-positive CYP had higher symptom prevalence. Mental health, well-being, fatigue and health-related quality of life were similar among test-positive and test-negative CYP, and symptoms at 6 months were similar in COVID-19 vaccinated and unvaccinated.

**Trial registration number:**

ISRCTN 34804192.

**WHAT IS ALREADY KNOWN ON THIS TOPIC**

- Few studies of long COVID in children and young people include a suitable comparison group.
- To our knowledge, no study has described self-reported overall health of children and young people 6 months after confirmed SARS-CoV-2 infection.
- No study has described post-COVID-19 symptoms in children and young people by vaccination status after proven SARS-CoV-2 infection.

**WHAT THIS STUDY ADDS**

- Six-months post-PCR testing, adolescents who tested positive for SARS-CoV-2 had similar symptoms to those who tested negative, but test-positives had a higher prevalence of symptoms.
- Applying a Delphi-derived research definition of long COVID, 24.5% of test-positive and 17.8% of test-negative children and young people would be classified as having long COVID at 6 months.
- Physical, mental health, fatigue and health-related quality of life were similar among COVID-19-vaccinated and COVID-19-unvaccinated test-positive and test-negative children and young people.

**HOW THIS STUDY MIGHT AFFECT RESEARCH, PRACTICE OR POLICY**

- Six months post-test, there was little difference in mental health between test-positive and test-negative adolescents, suggesting the impact on mental health of SARS-CoV-2 infection is small.
- A symptom-based definition of ‘long COVID’ may not be ideal, and more detailed phenotyping looking for changes in biomarkers, immunotype and imaging is needed.
- Data from this subsample suggest that a COVID-19 vaccination policy based on reducing long-term symptoms in adolescents might not be efficacious.

## Introduction

Research on the long-term physical and mental health of SARS-CoV-2 infection in children and young people (CYP), who have also suffered from the indirect effects of the pandemic, including school closures, education disruption and social isolation, is key.^1^

Our systematic review on persistent symptoms following SARS-CoV-2 in CYP found only 8 studies with an appropriate control group and 15 with an adequate follow-up period, mostly less than 4 months.^2^ Published reports on the natural history of long COVID and persistence of both physical and mental symptoms over time vary widely.^3–9^

The effect of COVID-19 vaccination on persistent symptoms in CYP is not known. In the UK, vaccination was recommended in August 2021 for healthy adolescents aged 16–17 years old and September 2021 for those 12–15 years old. By January 2022, 52.5% of adolescents aged 12–15 years old and 69.7% of those 16–17 years old in state-funded schools in England had received at least one dose of a COVID-19 vaccine, while 5.8% and 46.0%, respectively, received two doses.^10^

COVID-19 vaccination may reduce the risk of long COVID in adults who are subsequently infected with SARS-CoV-2.^11^ Furthermore, for pre-existing long COVID in adults, vaccination was associated with a 12.8% decrease in self-reported prevalence of long COVID; a second dose was associated with an 8.8% decrease.^12^

We describe the physical and mental health of adolescents aged 11–17 years old, 6 months after PCR testing for SARS-CoV-2 infection in those who tested positive and tested negative and explore variation by COVID-19 vaccination status using data from the CLoCk Study,^13^ the largest, prospective, matched cohort study of test-positive and test-negative CYP.

## Methods

The CLoCk Study^13^ is a national cohort study of SARS-CoV-2 PCR-positive (‘exposed’) CYP aged 11–17 years, matched at study invitation, by month of test, age, sex and geographical area to SARS-CoV-2 test-negative (‘unexposed’) CYP using the national SARS-CoV-2 testing dataset held by the UK Health Security Agency (UKHSA). UKHSA received results of all SARS-CoV-2 PCR tests in England irrespective of reason (school attendance, contact of positive case, symptomatic). Only UK National Health Service number, name, age, sex and postcode were recorded. UKHSA can access the electronic Patient Demographic Service allowing us to approach CYP by post for them to consent online and undertake our questionnaire. The CLoCk Study involves follow-up for 2 years after a SARS-CoV-2 PCR test taken September 2020–March 2021. Depending on month of test, for some participants this is at 3, 6, 12 and 24 months post-test; for others 6, 12 and 24 months post-test; and for some 12 and 24 months post-test. This paper reports on all 6-month data post-test. Approximately 25% of these participants had reported follow-up data at 3 months post-test (see the Results section for details).

### Measures

The questionnaire included demographics, elements of the International Severe Acute Respiratory and emerging Infection Consortium questionnaire,^14^ 21 symptoms, the EQ-Visual Analogue Scale^15^ and EQ-5D-Y^16^ scale, Strengths and Difficulties Questionnaire (SDQ),^17^ Short Warwick-Edinburgh Mental Wellbeing Scale (SWEMWBS)^18^ and Chalder Fatigue Scale^19^ (online supplemental text 1). Those who consented completed the online questionnaire describing their health at the time of PCR testing (baseline) and 6 months post-test. Hence, baseline data relating to health at PCR testing were collected retrospectively; data 6 months later were collected prospectively.

10.1136/archdischild-2022-324656.supp1 Supplementary data

CYP who were originally PCR negative but received a positive SARS-CoV-2 test by 6 months were excluded (determined by PCR test results held by UKHSA and self-report). Similarly, those who tested positive originally and were reinfected were also excluded. In addition, both test-positive and test-negative CYP were excluded if they responded to the 6-month questionnaire more than 34 weeks after their baseline PCR test.

### Statistical methods

The representativeness of our study population was assessed by comparing their demographics (sex, age, region of residence and Index of Multiple Deprivation (IMD)) with those of the target population (all invited participants). Baseline characteristics of study participants, symptoms reported at PCR testing and symptoms reported 6 months post-test were further assessed according to SARS-CoV-2 status. As the prevalence of long COVID might vary by age,^5 20^ we also stratified analyses into two age groups, reflecting key educational stages (11–14 and 15–17 years).^21^

We operationalised our Delphi research definition of long COVID^22^ as having at least 1 of 21 symptoms and experiencing more than minimal problems on any one of the five EQ-5D-Y questions^23^ (see footnote of online supplemental table 4). The Delphi definition requires laboratory confirmation of SARS-CoV-2 infection but of course that was not required when assessing how many test-negative CYP would also have met this definition. Participants’ characteristics at baseline and 6 months post-test were stratified by SARS-CoV-2 test status and then further stratified by their long COVID status. Similarly, we produced tables stratified by SARS-CoV-2 infection and COVID-19 vaccination status. There were missing data only for information on vaccination (an optional question at the time of questionnaire completion), where 20 (15 PCR-negative and 5 PCR-positive) CYP did not answer. These CYP were omitted from tables stratifying by vaccination status.

To assess the effect of potential response bias, we reweighted all symptom frequencies according to the age, sex, region, IMD and SARS-CoV-2 status of the respondents so that analyses align with the characteristics of the target population.

This is a descriptive study and, in line with guidance, significance tests were avoided.^24^ Therefore, our study does not assess causality nor report measures of association, and instead aims to describe distributions with the purpose of identifying areas for future formal investigation. This study is registered with the ISRCTN registry (ISRCTN 34804192).

## Results

**Figure 1:**
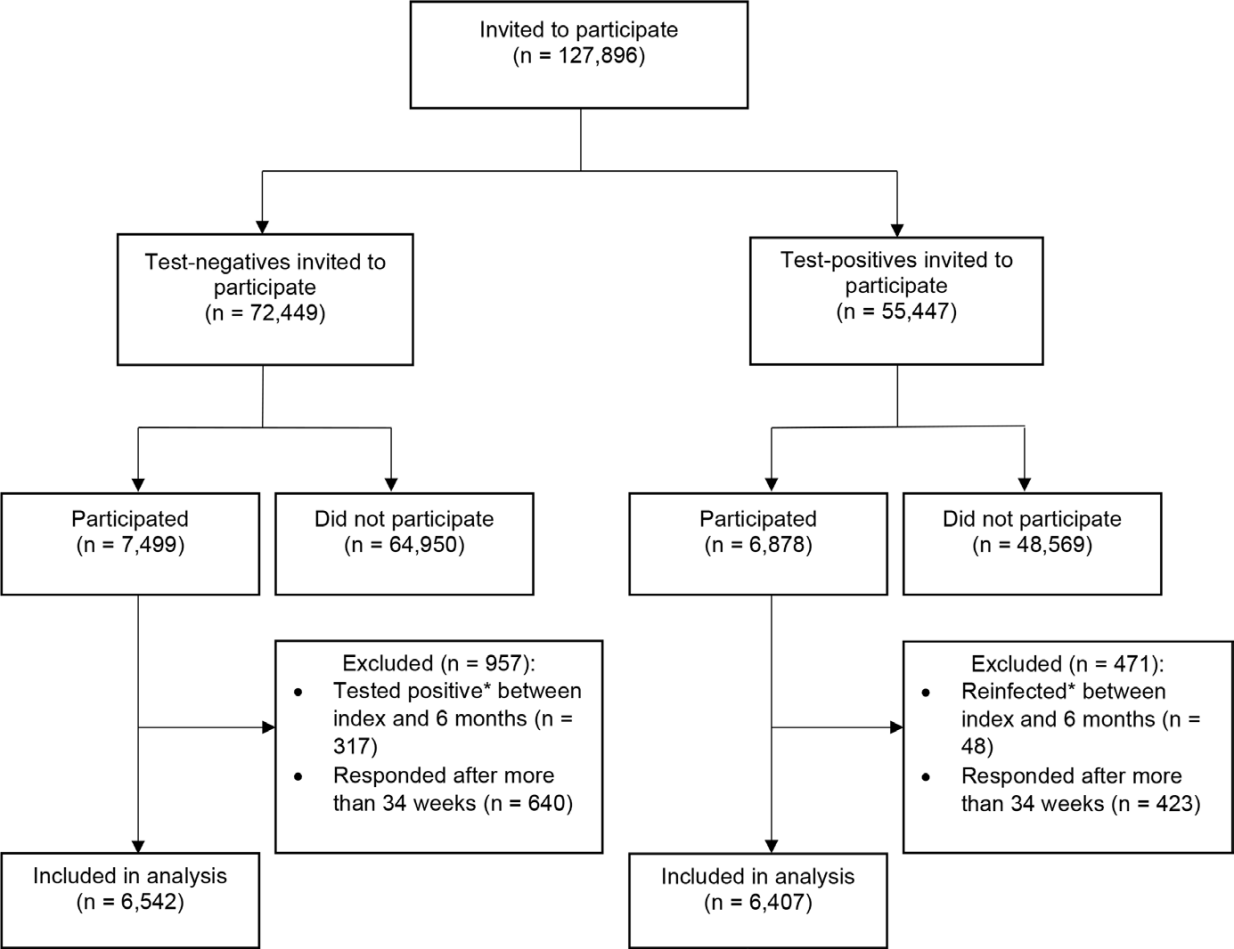
Participant flow. *Determined by PCR test results held by the UK Health Security Agency and self-report of whether (or not) the participant ever had a positive COVID-19 test.

A total of 14 377 participants completed a follow-up questionnaire 6 months after their SARS-CoV-2 PCR test between September 2020 and March 2021 (figure 1).^13^ Of these, 317 of 7499 CYP who were originally PCR negative received a positive SARS-CoV-2 test by 6 months and were excluded. Similarly, 48 of 6878 who tested positive originally were reinfected and excluded. An additional 1063 CYP (423 test positive and 640 test negative) were excluded because they responded to the 6-month questionnaire more than 34 weeks after their PCR test. The final study sample comprised 12 949 CYP (6407 tested positive, 6542 tested negative) and included 576 PCR-positive and 695 PCR-negative CYP who self-reported receiving a COVID-19 vaccine by their 6-month questionnaire. Although the study recruited non-hospitalised CYP at time of testing, 104 PCR-positive CYP did subsequently attend hospital appointments in relation to their COVID-19 infection of whom 50 were hospitalised overnight. A total of 1658 test-positive and 1737 test-negative participants who completed the questionnaire at 6 months had also completed the questionnaire at 3 months (when they reported on their baseline symptoms).^21^

The 6-month follow-up questionnaire was returned at a median of 27.8 (IQR: 26.3–29.7) weeks after testing. Overall, 6542 of 72 449 (9.0%) test-negative and 6407 of 55 447 (11.6%) test-positive CYP completing the 6-month questionnaire formed part of the analytical sample (see online supplemental table 1). Compared with the target population, both test-positive and test-negative respondents were more likely to be female and older teenagers (table 1). Study participants were also more likely to be from the East Midlands and the South West and less likely to be from London or the North West; they were also more likely to be from the least deprived areas. Test-negative and test-positive respondents in both the target population and analytical sample were broadly similar in terms of demographics, reflecting the matched cohort design.

**Table 1:** Demographics of target population and participants included in the 6-month sample

	Negative SARS-CoV-2 test		Positive SARS-CoV-2 test	
	Target population (N=72 449)	Study participants (N=6542)	Target population (N=55 447)	Study participants (N=6407)
Response rate		9.0%		11.6%
Sex				
Female	38 507 (53.2%)	4112 (62.9%)	29 443 (53.1%)	3992 (62.3%)
Male	33 942 (46.8%)	2430 (37.1%)	26 004 (46.9%)	2415 (37.7%)
Age (years)				
11–14	34 834 (48.1%)	2814 (43.0%)	26 757 (48.2%)	2759 (43.1%)
15–17	37 615 (51.9%)	3728 (57.0%)	28 690 (51.8%)	3648 (56.9%)
Ethnicity	Not recorded		Not recorded	
White		5083 (77.7%)		4919 (76.8%)
Asian, Asian British		856 (13.1%)		917 (14.3%)
Mixed		305 (4.7%)		265 (4.1%)
Black, African, Caribbean		172 (2.6%)		153 (2.4%)
Other		93 (1.4%)		112 (1.8%)
Prefer not to say		33 (0.5%)		41 (0.6%)
Region				
East Midlands	6232 (8.6%)	710 (10.9%)	4771 (8.6%)	643 (10.0%)
East of England	7273 (10.0%)	742 (11.3%)	5546 (10.0%)	649 (10.1%)
London	10 178 (14.0%)	824 (12.6%)	7950 (14.3%)	725 (11.3%)
North East England	4098 (5.7%)	379 (5.8%)	3079 (5.5%)	407 (6.4%)
North West England	13 590 (18.8%)	920 (14.1%)	10 363 (18.7%)	981 (15.3%)
South East England	8923 (12.3%)	890 (13.6%)	6816 (12.3%)	885 (13.8%)
South West England	4013 (5.5%)	489 (7.5%)	2934 (5.3%)	498 (7.8%)
West Midlands	9747 (13.5%)	877 (13.4%)	7386 (13.3%)	847 (13.2%)
Yorkshire and the Humber	8395 (11.6%)	711 (10.9%)	6602 (11.9%)	772 (12.1%)
IMD quintile*				
1 (most deprived)	21 584 (29.8%)	1286 (19.7%)	16 498 (29.8%)	1268 (19.8%)
2	14 737 (20.3%)	1175 (18.0%)	11 528 (20.8%)	1169 (18.3%)
3	12 546 (17.3%)	1220 (18.6%)	9589 (17.3%)	1120 (17.5%)
4	12 062 (16.6%)	1370 (20.9%)	9112 (16.4%)	1340 (20.9%)
5 (least deprived)	11 520 (15.9%)	1491 (22.8%)	8720 (15.7%)	1510 (23.6%)

*The IMD was calculated from the CYP’s small local area level-based geographical hierarchy (lower super output area) at the time of the questionnaire and used as a proxy for socioeconomic status. We report IMD quintiles from most (quintile 1) to least (quintile 5) deprived.

CYP, children and young people; IMD, Index of Multiple Deprivation.

### At testing

**Figure 2:**
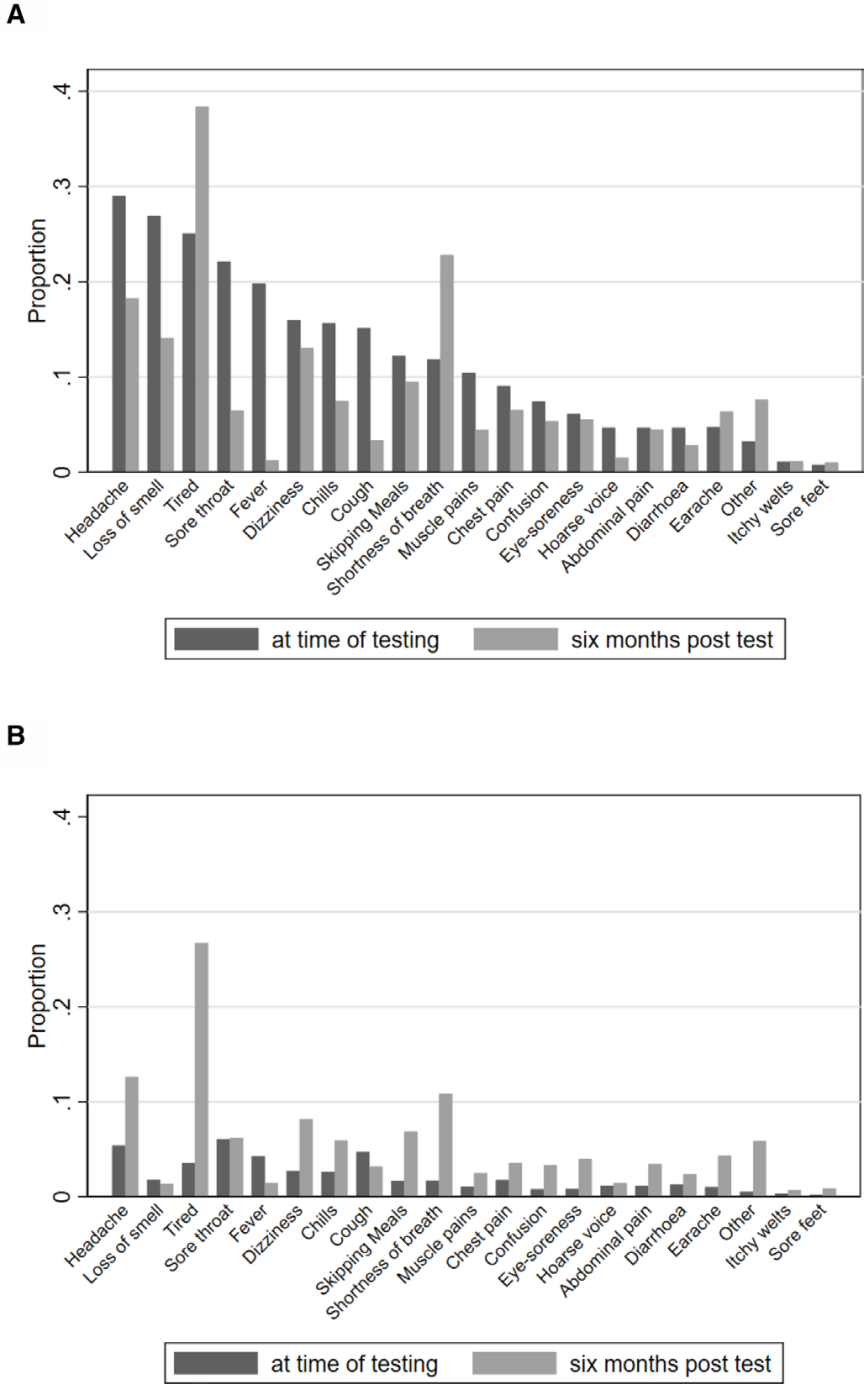
Proportion of participants experiencing symptoms at time of testing and 6 months post-testing by SARS-CoV-2 status: (A) test-positive children and young people (CYP) and (B) test-negative CYP.

At testing, test-positive CYP reported more symptoms than test-negative CYP (table 2). The most common symptoms among test-positive CYP were headaches, loss of smell and tiredness; among test-negative CYP they were sore throat, headaches and cough (table 2 and figure 2). The prevalence of symptoms varied by SARS-CoV-2 status (eg, headaches were reported by 29.0% of test-positive compared with 5.4% of test-negative CYP). The burden of symptoms was slightly higher at older ages (online supplemental table 2).

**Table 2:** Reported symptoms, and self-rated physical and mental health*, by SARS-CoV-2 status, at time of testing and 6 months post-test

	At time of testing		6 months post-test	
	Positive SARS-CoV-2 (n=6407)	Negative SARS-CoV-2 (n=6542)	Positive SARS-CoV-2 (n=6407)	Negative SARS-CoV-2 (n=6542)
No reported symptoms	3736 (58.3%)	5921 (90.5%)	2507 (39.1%)	3713 (56.8%)
1 symptom	239 (3.7%)	77 (1.2%)	1355 (21.2%)	1228 (18.8%)
2 symptoms	262 (4.1%)	94 (1.4%)	779 (12.2%)	563 (8.6%)
3 symptoms	297 (4.6%)	98 (1.5%)	569 (8.9%)	318 (4.9%)
4 symptoms	306 (4.8%)	83 (1.3%)	393 (6.1%)	216 (3.3%)
≥5 symptoms	1567 (24.5%)	269 (4.1%)	804 (12.6%)	504 (7.7%)
Specific symptoms				
Fever	1269 (19.8%)	279 (4.3%)	82 (1.3%)	96 (1.5%)
Chills	1002 (15.6%)	173 (2.6%)	479 (7.5%)	387 (5.9%)
Persistent cough	969 (15.1%)	310 (4.7%)	215 (3.4%)	209 (3.2%)
Tiredness	1607 (25.1%)	233 (3.6%)	2458 (38.4%)	1747 (26.7%)
Shortness of breath	759 (11.9%)	112 (1.7%)	1462 (22.8%)	710 (10.9%)
Loss of smell	1726 (26.9%)	117 (1.8%)	903 (14.1%)	90 (1.4%)
Unusually hoarse voice	299 (4.7%)	77 (1.2%)	98 (1.5%)	95 (1.5%)
Unusual chest pain	580 (9.1%)	116 (1.8%)	419 (6.5%)	234 (3.6%)
Unusual abdominal pain	299 (4.7%)	76 (1.2%)	288 (4.5%)	226 (3.5%)
Diarrhoea	299 (4.7%)	85 (1.3%)	181 (2.8%)	157 (2.4%)
Headaches	1858 (29.0%)	353 (5.4%)	1171 (18.3%)	825 (12.6%)
Confusion, disorientation or drowsiness	476 (7.4%)	53 (0.8%)	345 (5.4%)	218 (3.3%)
Unusual eye soreness	393 (6.1%)	54 (0.8%)	356 (5.6%)	260 (4.0%)
Skipping meals	782 (12.2%)	111 (1.7%)	609 (9.5%)	448 (6.9%)
Dizziness or light-headedness	1023 (16.0%)	180 (2.8%)	838 (13.1%)	535 (8.2%)
Sore throat	1418 (22.1%)	397 (6.1%)	414 (6.5%)	406 (6.2%)
Unusual strong muscle pains	670 (10.5%)	69 (1.1%)	286 (4.5%)	163 (2.5%)
Earache or ringing in ears	304 (4.7%)	66 (1.0%)	407 (6.4%)	284 (4.3%)
Raised welts on skin or swelling	71 (1.1%)	21 (0.3%)	73 (1.1%)	47 (0.7%)
Red/purple sores/blisters on feet	50 (0.8%)	14 (0.2%)	66 (1.0%)	58 (0.9%)
Other	209 (3.3%)	35 (0.5%)	488 (7.6%)	384 (5.9%)
Previous physical health*				
Very poor or poor	107 (1.7%)	148 (2.3%)		
Okay	1212 (18.9%)	1428 (21.8%)		
Good or very good	5088 (79.4%)	4966 (75.9%)		
Previous mental health*				
Very poor or poor	516 (8.1%)	667 (10.2%)		
Okay	1790 (27.9%)	1968 (30.1%)		
Good or very good	4101 (64.0%)	3907 (59.7%)		
Self-rated health†	90 (85–100)	90 (80–100)	90 (80–95)	90 (80–100)

Data are n (%).

*Participants were asked ‘How was your physical/mental health in general before your COVID-19 test?’ in two separate questions using a five-category Likert scale; we recoded these variables into three categories (very poor and poor, okay, and good and very good); questions were not asked in relation to 6 months post-test.

†Reported as median (IQR), measured by a Visual Analogue Scale (VAS) (EQ-5D VAS score), which records responses to the question ‘Please look at the scale and select the number for your health BEFORE your COVID-19 test and your health TODAY’. Participants were told that ‘100% means the best health you can think of; 0% means the worst health you can think of’.

### Six months post-testing

Six months post-testing, the most common symptoms in test-positive CYP were tiredness, shortness of breath, headaches, loss of smell and dizziness in that order (table 2 and figure 2); all other symptoms affected less than 10% of test-positive CYP. The most common symptoms in test-negative CYP were tiredness, headaches and shortness of breath in that order; all other symptoms affected less than 10% of test-negative CYP. The prevalence of individual symptoms was higher in the test-positive CYP (table 2) and in the older age group (online supplemental table 3). Notably, 6 months post-test, despite the higher prevalence of symptoms in test-positive CYP compared with test-negative CYP, self-rated health was similar in both groups, overall and when stratified by age (online supplemental table 3).

**Figure 3:**
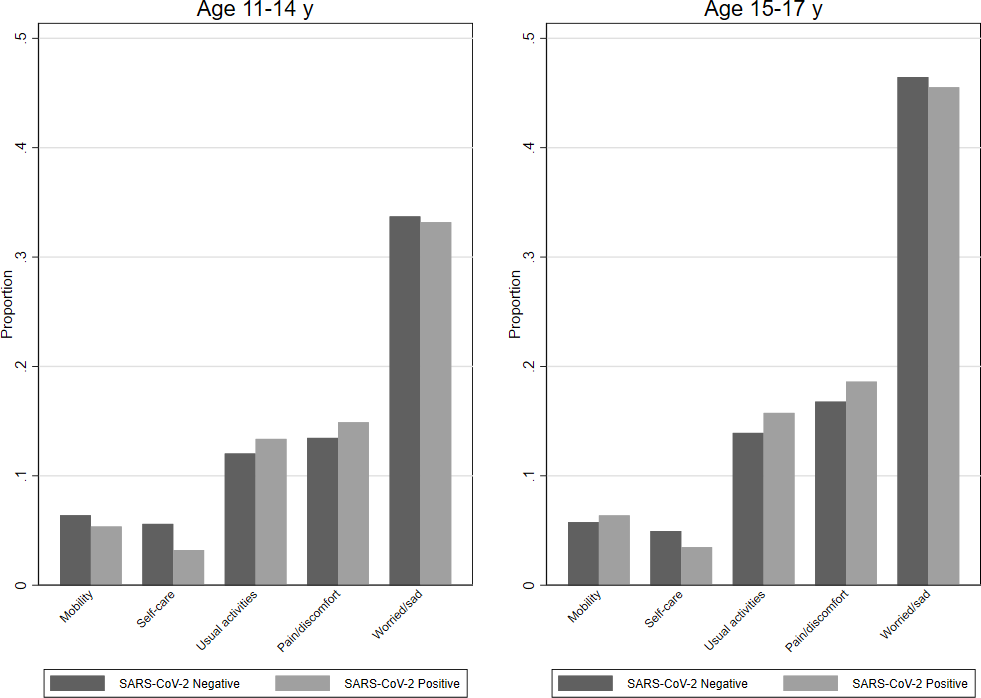
Proportion of participants reporting experiencing some or a lot of problems* 6 months post-test by SARS-CoV-2 status, by age. *Assessed using the five items from the EQ-5D-Y scale, each of which has a three-level response (no problems, some problems, a lot of problems). The graph shows the proportion who experiences some/a lot of problems with mobility, self-care, doing usual activities, having pain/discomfort, or feeling a bit/very worried/sad.

Six months post-test, mental health and well-being were similar between test-positive and test-negative CYP. Among those aged 11–14 years old, the SDQ (total difficulties) median was 9 (IQR 5–14) for test-positive and 10 (IQR 6–15) for test-negative CYP (higher SDQ scores indicate more problems). Among those aged 15–17 years old, the SDQ median was 11 (IQR 7–16) for test-positive and 12 (IQR 7–17) for test-negative CYP. The distribution of SWEMWBS scores (higher scores indicate better mental well-being) was similar between the test-positive (mean=21.7; SD 4.3) and the test-negative CYP (mean=21.4; SD 4.3). Mean fatigue scores (higher scores indicate more severe fatigue) were also similar between the test-positive (13.4; SD 5.1) and test-negative CYP (13.0; SD 5.1). EQ-5D-Y scores, representing health-related quality of life, showed that test-positive and test-negative CYP were equally likely to report problems with mobility, self-care, doing usual activities, pain/discomfort and feeling worried/sad, for example, 45.5% of test-positive CYP aged 15–17 years old, and 46.5% of test-negative CYP felt worried, sad or unhappy, as indicated on a single item of the EQ-5D-Y (figure 3).

Six months post-test, 24.5% of test-positive and 17.8% of test-negative CYP met our Delphi research definition of long COVID^22^ (online supplemental table 4). Regardless of SARS-CoV-2 status, those meeting the research definition of long COVID at 6 months also reported worse mental health, well-being and fatigue (online supplemental table 5).

Between testing and 6 months post-test, 10.7% of test-negative and 9.0% of test-positive CYP received a COVID-19 vaccine (online supplemental table 6). When assessed by SARS-CoV-2 PCR test status at baseline, there was little difference between vaccinated and unvaccinated CYP in terms of symptoms, mental health or well-being at 6 months post-test (online supplemental table 7).

When we reweighted the percentage of reported symptoms at baseline and at 6 months post-test according to the age, sex, region, IMD and SARS-CoV-2 test status of the respondents, so that analyses align with the characteristics of the target population, broadly similar patterns were observed to those reported above (online supplemental table 8).

## Discussion

At time of testing for SARS-CoV-2 infection, test-positive CYP reported more symptoms than test-negative CYP. Six months post-testing, the overall prevalence of symptoms had increased in both groups and the prevalence of symptoms remained higher in test-positive compared with test-negative CYP. As in our previous analysis at 3 months post-test,^21^ prevalence of tiredness and shortness of breath increased at 6 months post-test compared with baseline in both test-positive and test-negative CYP. It is these two large increases which underlie the overall increase in symptom prevalence in both groups. This may be partly due to self-selection by participants with symptoms to report. Study participants were more likely to be female, older, from certain geographical areas and from less deprived areas.

Employing our Delphi consensus definition of long COVID in CYP,^22^ those without long COVID are more likely to be male, younger, and have good/very good physical and mental health before PCR testing (online supplemental table 4).

With respect to mental health, well-being and fatigue 6 months post-test, there was little difference in scores between test-positive and test-negative CYP. Moreover, the scores were very similar to those we described at 3-month follow-up.^21^ For example, at both 3 and 6 months post-test, median SDQ was 9–12 depending on age.

It might appear incongruent that 6 months post-test, while test-positive CYP had a higher prevalence of symptoms than test-negative CYP, their mental health, self-rated health, well-being and fatigue levels were similar. These observations suggest that by 6 months post-test, while test-positive CYP do experience more symptoms than test-negative CYP, these symptoms are mostly mild with little effect on overall well-being.

Acknowledging only 10% of CYP were vaccinated by 6 months post-test, we found little difference in symptoms, mental health or well-being at 6 months in vaccinated and unvaccinated PCR-positive and PCR-negative CYP. Indeed, EQ-5D-Y scores were somewhat worse and self-rated health lower, for the vaccinated group, which could represent self-selection of more severely affected CYP undergoing vaccination or self-selection of respondents.

The very few studies with follow-up of CYP for at least 6 months after SARS-COV-2 infection^4 6 7^ show conflicting findings. However, all findings, including ours, need to be considered within the context of bias (selection and recall), low response rates and recognising that a temporal association with infection does not prove causality as indirect effects of the pandemic need to be considered.^25^ Therefore, our findings are important because we have a test-negative group of CYP who have lived through the ‘long pandemic’ and who never tested positive for SARS-CoV-2 before or during the study period, although we acknowledge that (re)infections may have gone undetected. However, PCR and lateral flow tests were widely and freely available in the UK before and during the 6 months post-test period (April–September 2021). Hence, our data provide a unique perspective on long COVID in CYP.

Our study has limitations.^21^ First, the questionnaire response rate was low (9.0% (test-negative CYP); 11.6% (test-positive CYP)). However, this is similar to the Office for National Statistics random household survey (response rate 12% October 2021, Daniel Ayoubkhani: personal communication). Second, the study design may risk selection biases of those with internet access; symptoms to report (perhaps explaining why relative frequencies of many symptoms were higher 6 months post-test compared with baseline); recall bias for symptoms at time of testing; returning to school from March 2021, following national lockdown from January 2021, with exposure to other infections. Furthermore, some symptoms might have predated SARS-CoV-2 infection. Participants did not report on symptom severity. Third, although the number of symptoms is a proxy of illness severity, a single severe symptom might be more disabling than several mild symptoms. EQ-5D-Y served as a severity indicator because it assesses the effect on daily living. Fourth, it is possible that some participants might have been misdiagnosed as SARS-CoV-2 negative and vice-versa. False negatives can arise from PCR timing, swab technique and assay sensitivity but false-positive PCR results are rare. Fifth, we could not recruit or match on ethnicity, medical history or testing location but subsequent self-reported ethnicity was very similar in both test groups and geographical address served as a proxy for socioeconomic status. We used established scales to measure mental health, well-being and fatigue but acknowledge the limitations of self-reporting and floor and ceiling effects.

All self-reported symptoms are subjective. Researchers want to ask as much as possible to allow analysis of as many research questions as possible. In our pilot, the researchers’ initial draft questionnaire took over an hour to complete and teenagers said they would be willing to spend 20 minutes maximum completing the survey, despite a financial reward. This compromise means our wide-ranging and unique data have limitations in terms of information available.

## Conclusions

Tiredness and shortness of breath were two dominant symptoms 6 months after SARS-CoV-2 PCR testing, irrespective of test result. Secondly, 27.6% of test-positive and 15.9% of test-negative CYP had three or more physical symptoms 6 months post-test, which was similar to the 30.3% and 16.2%, respectively, we reported at 3 months post-test.^21^ Thirdly, at 6 months post-test, there was little difference in well-being and mental health between test-positive and test-negative CYP. Fourth, 24.5% of test-positive CYP met our Delphi definition of long COVID^22^ at 6 months compared with 17.8% of test-negative CYP. Finally, the profile of symptoms, well-being and mental health in test-positive and test-negative CYP by COVID-19 vaccination status was similar.

## References

[1] World Health Organization . WHO/Europe | Child and adolescent health - COVID-19 and Children, 2022. Available: https://www.euro.who.int/en/health-topics/Life-stages/child-and-adolescent-health/covid-19-and-children [Accessed 06 Jun 2022].

[2] Behnood SA , Shafran R , Bennett SD , et al . Persistent symptoms following SARS-CoV-2 infection amongst children and young people: a meta-analysis of controlled and uncontrolled studies. J Infect 2022;84:158–70. 10.1016/j.jinf.2021.11.011 34813820 PMC8604800

[3] Castanares-Zapatero Diego KL , Marie D , Jens D , et al . Long COVID: pathophysiology – epidemiology and patient needs. health services research (hsr. Brussels.: Belgian Health Care Knowledge Centre (KCE), 2021.

[4] Say D , Crawford N , McNab S , et al . Post-acute COVID-19 outcomes in children with mild and asymptomatic disease. Lancet Child Adolesc Health 2021;5:e22–3. 10.1016/S2352-4642(21)00124-3 33891880 PMC8057863

[5] Molteni E , Sudre CH , Canas LS , et al . Illness duration and symptom profile in symptomatic UK school-aged children tested for SARS-CoV-2. Lancet Child Adolesc Health 2021;5:708–18. 10.1016/S2352-4642(21)00198-X 34358472 PMC8443448

[6] Osmanov IM , Spiridonova E , Bobkova P , et al . Risk factors for post-COVID-19 condition in previously hospitalised children using the ISARIC global follow-up protocol: a prospective cohort study. Eur Respir J 2022;59:2101341. 10.1183/13993003.01341-2021 34210789 PMC8576804

[7] Magnusson K , Skyrud KD , Suren P , et al . Healthcare use in 700 000 children and adolescents for six months after covid-19: before and after register based cohort study. BMJ 2022;376:e066809. 10.1136/bmj-2021-066809 35039315 PMC8762452

[8] Borch L , Holm M , Knudsen M , et al . Long COVID symptoms and duration in SARS-CoV-2 positive children - a nationwide cohort study. Eur J Pediatr 2022;181:1597–607. 10.1007/s00431-021-04345-z 35000003 PMC8742700

[9] Kikkenborg Berg S , Dam Nielsen S , Nygaard U , et al . Long COVID symptoms in SARS-CoV-2-positive adolescents and matched controls (LongCOVIDKidsDK): a national, cross-sectional study. Lancet Child Adolesc Health 2022;6:240–8. 10.1016/S2352-4642(22)00004-9 35143771 PMC8820960

[10] GOV.UK . Vaccinations in the UK | coronavirus in the UK, 2022. Available: https://coronavirus.data.gov.uk/details/vaccinations [Accessed 06 Jun 2022].

[11] Antonelli M , Penfold RS , Merino J , et al . Risk factors and disease profile of post-vaccination SARS-CoV-2 infection in UK users of the COVID symptom study APP: a prospective, community-based, nested, case-control study. Lancet Infect Dis 2022;22:43–55. 10.1016/S1473-3099(21)00460-6 34480857 PMC8409907

[12] Ayoubkhani D , Bermingham C , Pouwels KB , et al . Trajectory of long covid symptoms after covid-19 vaccination: community based cohort study. BMJ 2022;377:e069676. 10.1136/bmj-2021-069676 35584816 PMC9115603

[13] Stephenson T , Shafran R , De Stavola B , et al . Long COVID and the mental and physical health of children and young people: national matched cohort study protocol (the clock study). BMJ Open 2021;11:e052838. 10.1136/bmjopen-2021-052838 PMC8392739 34446502

[14] Sigfrid L , Buonsenso D , Galvin A , et al . ISARIC global COVID-19 paediatric follow-up, 2021. Available: https://isaric.org/research/covid-19-clinical-research-resources/paediatric-follow-up/ [Accessed 02 Nov 2021].

[15] Devlin N , Parkin D , Janssen B . Chapter 3 Analysis of EQ VAS Data. In: Devlin N , Parkin D , Janssen B , eds. Methods for Analysing and Reporting EQ-5D Data [Internet]. Cham: Springer, 2020. 33347096

[16] Wille N , Badia X , Bonsel G , et al . Development of the EQ-5D-Y: a child-friendly version of the EQ-5D. Qual Life Res 2010;19:875–86. 10.1007/s11136-010-9648-y 20405245 PMC2892611

[17] Goodman R . Psychometric properties of the strengths and difficulties questionnaire. J Am Acad Child Adolesc Psychiatry 2001;40:1337–45. 10.1097/00004583-200111000-00015 11699809

[18] Tennant R , Hiller L , Fishwick R , et al . The Warwick-Edinburgh mental well-being scale (WEMWBS): development and UK validation. Health Qual Life Outcomes 2007;5:63. 10.1186/1477-7525-5-63 18042300 PMC2222612

[19] Chalder T , Berelowitz G , Pawlikowska T , et al . Development of a fatigue scale. J Psychosom Res 1993;37:147–53. 10.1016/0022-3999(93)90081-P 8463991

[20] Office for National Statistics . Prevalence of ongoing symptoms following coronavirus (COVID-19) infection in the UK statistical bulletins, 2022. Available: https://www.ons.gov.uk/peoplepopulationandcommunity/healthandsocialcare/conditionsanddiseases/bulletins/prevalenceofongoingsymptomsfollowingcoronaviruscovid19infectionintheuk/previousReleases [Accessed 01 Jul 2022].

[21] Stephenson T , Pinto Pereira SM , Shafran R , et al . Physical and mental health 3 months after SARS-CoV-2 infection (long COVID) among adolescents in England (clock): a national matched cohort study. Lancet Child Adolesc Health 2022;6:230–9. 10.1016/S2352-4642(22)00022-0 35143770 PMC8820961

[22] Stephenson T , Allin B , Nugawela MD , et al . Long COVID (post-COVID-19 condition) in children: a modified Delphi process. Arch Dis Child 2022;107:674–80. 10.1136/archdischild-2021-323624 35365499 PMC8983414

[23] Nugawela MD , Stephenson T , Shafran R , et al . Developing a model for predicting impairing physical symptoms in children 3 months after a SARS-CoV-2 PCR-test: the clock study. medRxiv 2022. 10.1101/2022.04.01.22273117

[24] Vandenbroucke JP , von Elm E , Altman DG , et al . Strengthening the reporting of observational studies in epidemiology (STROBE): explanation and elaboration. Ann Intern Med 2007;147:W–94. 10.7326/0003-4819-147-8-200710160-00010-w1 17938389

[25] Rytter MJH . Difficult questions about long COVID in children. Lancet Child Adolesc Health 2022;6:595–7. 10.1016/S2352-4642(22)00167-5 35752193 PMC9221929

