# Supplementary material for: Post-COVID-19 condition at 6 months and COVID-19 vaccination in non-hospitalised children and young people
Source: Arch Dis Child. 2023 Jan 4;108(4):289–95. doi: 10.1136/archdischild-2022-324656 (PMC10086284; doi:10.1136/archdischild-2022-324656)
Supplement: Supplementary data [file archdischild-2022-324656supp001.pdf]

Supplementary Files

Table of Contents

**Content**	**Page(s)**
Supplementary Text 1: CloCk Questionnaire	2-7
Supplementary Table 1	8
Supplementary Table 2	9-10
Supplementary Table 3	11
Supplementary Table 4	12-13
Supplementary Table 5	14
Supplementary Table 6	15-16
Supplementary Table 7	17-18
Supplementary Table 8	19-21

Pinto Pereira SM, *et al. Arch Dis Child* 2023; 108:289–295. doi: 10.1136/archdischild-2022-324656

About you

How tall are you? _______________ (❑ cm ❑ metres ❑ feet/inches) ❑ Not sure

What is your weight now? _______________ (❑ kg ❑ stone ❑ lbs) ❑ Not sure

About your COVID-19 test

Have you had a COVID-19 test since the last time you completed this questionnaire?
❑ Yes ❑ No
How many COVID-19 tests have you had? _______________
When was your COVID-19 test? (if more than 1: please enter date of your first COVID-19 test)
[_D_][_D_]/[_M_][_M_]/[_2_][_0_][_Y_][_Y_]
If more than 1: When was your most recent COVID-19 test?
[_D_][_D_]/[_M_][_M_]/[_2_][_0_][_Y_][_Y_]

What was the result? (please answer in relation to your last COVID-19 test)
❑ Positive ❑ Negative
If your test was positive, is this your first positive result? ❑ Yes ❑ No
If your test was negative, do you believe that you had COVID-19? (please answer these in relation to your last Covid-19 test) ❑ Yes ❑ No ❑ Not sure ❑ Not applicable
What was the reason for your most recent COVID-19 test? (please answer these in relation to your last COVID-19 test)
❑ I had symptoms ❑ I was near someone who had tested positive ❑ School testing ❑ Other
In the last four weeks, how many school days (online or in person) in total did you miss because of symptoms of COVID-19
❑ None ❑ 1-2 days ❑ 3-5 days ❑ 6-10 days ❑ 11-15 days ❑ More than 15 days

If you had symptoms please answer the following questions
When did you first notice them? (please answer these in relation to your last COVID-19 test)
[_D_][_D_]/[_M_][_M_]/[_2_][_0_][_Y_][_Y_]
How long did they last? (please answer these in relation to your last COVID-19 test)
❑ A day or less ❑ a few days ❑ about a week ❑ more than a week ❑ a couple of weeks or more
How bad were the symptoms at their worst? (please answer these in relation to your last COVID-19 test)
❑ Not very – I could carry on doing things ❑ a little – I felt a little bit poorly
❑ quite bad – I had to go to bed sometimes ❑ very bad – I couldn’t do much
❑ Extremely bad – I couldn’t do anything
What symptoms did you have? (Check all that apply) (please answer these in relation to your last COVID-19 test)
❑ fever
❑ chills or shivers (feeling too cold)
❑ persistent cough (coughing a lot for more than an hour, or 3 or more coughing episodes in 24 hours)
❑ unusual fatigue/tiredness
❑ unusual shortness of breath
❑ loss of smell/taste
❑ unusually hoarse voice
❑ unusual chest pain or tightness in your chest
❑ unusual abdominal pain
❑ diarrhoea
❑ headache

- ❑ confusion, disorientation or drowsiness
- ❑ unusual eye-soreness or discomfort (e.g. light sensitivity, excessive tears, or pink/red eye)
- ❑ skipping meals
- ❑ dizziness or light-headedness
- ❑ sore throat
- ❑ unusual strong muscle pains
- ❑ earache or ringing in your ears (tinnitus)
- ❑ raised, red, itchy welts on the skin or sudden swelling of the face or lips
- ❑ red/purple sores or blisters on your feet, including your toes
- ❑ other

If other, please state

What were your main symptoms? (please answer these in relation to your last COVID-19 test)

- ❑ fever
- ❑ chills or shivers (feeling too cold)
- ❑ persistent cough (coughing a lot for more than an hour, or 3 or more coughing episodes in 24 hours)
- ❑ unusual fatigue/tiredness
- ❑ unusual shortness of breath
- ❑ loss of smell/taste
- ❑ unusually hoarse voice
- ❑ unusual chest pain or tightness in your chest
- ❑ unusual abdominal pain
- ❑ diarrhoea
- ❑ headache
- ❑ confusion, disorientation or drowsiness
- ❑ unusual eye-soreness or discomfort (e.g. light sensitivity, excessive tears, or pink/red eye)
- ❑ skipping meals
- ❑ dizziness or light-headedness
- ❑ sore throat
- ❑ unusual strong muscle pains
- ❑ earache or ringing in your ears (tinnitus)
- ❑ raised, red, itchy welts on the skin or sudden swelling of the face or lips
- ❑ red/purple sores or blisters on your feet, including your toes
- ❑ other

If other, please state

Did you/your parent talk to the doctor about your COVID-19 symptoms? (please answer these in relation to your last COVID-19 test) ❑ Yes ❑ No

Did you go to the hospital about your COVID-19? (please answer these in relation to your last COVID-19 test)

❑ Yes ❑ No

Did you have to stay overnight in hospital for COVID-19? (please answer these in relation to your last COVID-19 test)

❑ Yes ❑ No

Have you had a vaccination against COVID-19?

Pinto Pereira SM, *et al. Arch Dis Child* 2023; 108:289–295. doi: 10.1136/archdischild-2022-324656

❑ Yes ❑ No

About your health at the moment

If you have had symptoms of COVID-19 how much do you agree with the following statement?
"I have fully recovered from COVID-19"

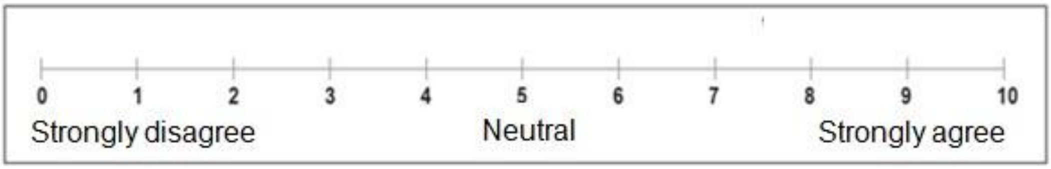

How do you feel right now? ❑ I feel as healthy as normal ❑ I am not feeling quite right
Do you have a fever? ❑ Yes ❑ No

Do you feel chills or shivers (feel too cold)? ❑ Yes ❑ No
If you are able to measure it, what is your temperature?
____

Do you have a persistent cough (coughing a lot for more than an hour, or 3 or more coughing episodes in 24 hours)?
❑ Yes ❑ No
Are you experiencing unusual fatigue/tiredness?
❑ No ❑ Mild fatigue ❑ Severe fatigue - I struggle to get out of bed
Are you experiencing problems with your sleep, including getting to sleep, waking in the night or waking early?
❑ Yes ❑ No
If yes, please describe
____

Are you experiencing unusual shortness of breath?
❑ No
❑ Yes, mild symptoms - slight shortness of breath during ordinary activity
❑ Yes, significant symptoms - breathing is comfortable only at rest
❑ Yes, severe symptoms - breathing is difficult even at rest
What are your current symptoms? (Please tick all that apply)
❑ loss of smell/taste
❑ unusually hoarse voice
❑ unusual chest pain or tightness in your chest
❑ unusual abdominal pain
❑ diarrhoea
❑ headache
❑ confusion, disorientation or drowsiness
❑ unusual eye-soreness or discomfort (e.g. light sensitivity, excessive tears, or pink/red eye)
❑ skipping meals
❑ dizziness or light-headedness
❑ sore throat
❑ unusual strong muscle pains

- ❑ earache or ringing in your ears (tinnitus)
- ❑ raised, red, itchy welts on the skin or sudden swelling of the face or lips
- ❑ red/purple sores or blisters on your feet, including your toes
- ❑ no symptoms
- ❑ other

Are there any other important symptoms you want to share

Since the start of your COVID-19 symptoms, have you had a period longer than one week with none of the above symptoms at all (where you were back to how you were pre-COVID)

- ❑ Yes (I have had a period of one week or more since my test with none of the above symptoms)
- ❑ No (My symptoms have been continuous since Covid test)
- ❑ Not applicable

Standarised Scales

EQ-5D-Y
Short Warwick-Edinburgh Mental Wellbeing Scale
Strengths and Difficulties Questionnaire © Robert Goodman, 2005
Chalder Fatigue Scale
UCLA 3 item loneliness scale

We would like to know how good or bad your health is TODAY
This scale is numbered from 0 to 100%
100% means the best health you can think of
0% means the worst health you can think of.

Please look at the scale and draw a circle to select the number for your health TODAY

Today

Pinto Pereira SM, *et al. Arch Dis Child* 2023; 108:289–295. doi: 10.1136/archdischild-2022-324656

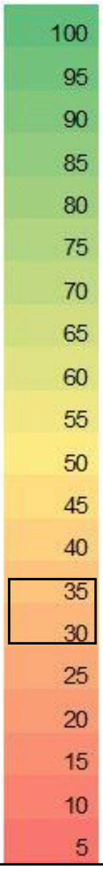

COVID-19 and your family

Has Covid-19 affected your family members and if so, can you tell us who?

	In your house				In your extended family			
	Yes	No	Don't know	Who?	Yes	No	Don't know	Who?
Has anyone tested positive for Covid-19?								
Has anyone been to hospital with Covid-19?								
Has anyone been in intensive care (ICU) with Covid-19?								
Has anyone died from Covid-19?								
Does anyone have ongoing problems from Covid-19?								

Final Question

Pinto Pereira SM, *et al. Arch Dis Child* 2023; 108:289–295. doi: 10.1136/archdischild-2022-324656

Please use this space if there is there anything else you would like to tell us about your health or how the pandemic or lockdown have affected you.

Supplement Table 1. Response rate of participants included in the 6-month sample

	Negative SARS-CoV-2 test		Positive SARS-CoV-2 test	
	Target Population (N=72,449)	Study participants (N=6,542)	Target Population (N=55,447)	Study participants (N=6,407)
Response rate		9.0%		11.6%
**Sex**				
Female	38,507	4,112 (10.7%)	29,443	3,992 (13.6%)
Male	33,942	2,430 (7.2%)	26,004	2,415 (9.3%)
**Age (years)**				
11-14	34,834	2,814 (8.1%)	26,757	2,759 (10.3%)
15-17	37,615	3,728 (9.9%)	28,690	3,648 (12.7%)
**Region**				
East Midlands	6,232	710 (11.4%)	4,771	643 (13.5%)
East of England	7,273	742 (10.2%)	5,546	649 (11.7%)
London	10,178	824 (8.1%)	7,950	725 (9.1%)
North East England	4,098	379 (9.2%)	3,079	407 (13.2%)
North West England	13,590	920 (6.8%)	10,363	981 (9.5%)
South East England	8,923	890 (10.0%)	6,816	885 (13.0%)
South West England	4,013	489 (12.2%)	2,934	498 (17.0%)
West Midlands	9,747	877 (9.0%)	7,386	847 (11.5%)
Yorkshire and the Humber	8,395	711 (8.5%)	6,602	772 (11.7%)
**IMD quintile***				
1 (most deprived)	21,584	1,286 (6.0%)	16,498	1,268 (7.7%)
2	14,737	1,175 (8.0%)	11,528	1,169 (10.1%)
3	12,546	1,220 (9.7%)	9,589	1,120 (11.7%)
4	12,062	1,370 (11.4%)	9,112	1,340 (14.7%)
5 (least deprived)	11,520	1,491 (12.9%)	8,720	1,510 (17.3%)

*IMD: Index of Multiple Deprivation. The Index of Multiple Deprivation (IMD) was calculated from the CYP's small local area level based geographic hierarchy (lower super output area) at the time of the questionnaire and used as a proxy for socio-economic status. We report IMD quintiles from most (quintile 1) to least (quintile 5) deprived.

Supplement Table 2. Reported symptoms at time of test, and physical and mental health before test, by SARS-CoV-2 status, overall and stratified by age group

	All participants		*Participants aged 11–14 years*		*Participants aged 15–17 years*	
	Tested positive for SARS-CoV-2 (n=6,407)	Tested negative for SARS-CoV-2 (n = 6,542)	Tested positive for SARS-CoV-2 (n=2,759)	Tested negative for SARS-CoV-2 (n=2,814)	Tested positive for SARS-CoV-2 (n=3,648)	Tested negative for SARS-CoV-2 (n=3,728)
**No reported symptoms**	3,736 (58.3%)	5,921 (90.5%)	1,626 (58.9%)	2,499 (88.8%)	2,110 (57.8%)	3,422 (91.8%)
1 symptom	239 (3.7%)	77 (1.2%)	107 (3.9%)	55 (2.0%)	132 (3.6%)	22 (0.6%)
2 symptoms	262 (4.1%)	94 (1.4%)	135 (4.9%)	56 (2.0%)	127 (3.5%)	38 (1.0%)
3 symptoms	297 (4.6%)	98 (1.5%)	140 (5.1%)	52 (1.9%)	157 (4.3%)	46 (1.2%)
4 symptoms	306 (4.8%)	83 (1.3%)	151 (5.5%)	47 (1.7%)	155 (4.3%)	36 (1.0%)
≥5 symptoms	1,567 (24.5%)	269 (4.1%)	600 (21.8%)	105 (3.7%)	967 (26.5%)	164 (4.4%)
**Specific symptoms**						
Fever	1,269 (19.8%)	279 (4.3%)	561 (20.3%)	129 (4.6%)	708 (19.4%)	150 (4.0%)
Chills	1,002 (15.6%)	173 (2.6%)	407 (14.7%)	66 (2.4%)	595 (16.3%)	107 (2.9%)
Persistent cough	969 (15.1%)	310 (4.7%)	358 (12.9%)	156 (5.5%)	611 (16.8%)	154 (4.1%)
Tiredness	1,607 (25.1%)	233 (3.6%)	622 (22.5%)	100 (3.6%)	985 (27.0%)	133 (3.6%)
Shortness of breath	759 (11.9%)	112 (1.7%)	211 (7.7%)	38 (1.4%)	548 (15.0%)	74 (2.0%)
Loss of smell	1,726 (26.9%)	117 (1.8%)	620 (22.4%)	53 (1.9%)	1,106 (30.3%)	64 (1.7%)
Unusually hoarse voice	299 (4.7%)	77 (1.2%)	114 (4.1%)	38 (1.4%)	185 (5.1%)	39 (1.1%)
Unusual chest pain	580 (9.1%)	116 (1.8%)	169 (6.1%)	45 (1.6%)	411 (11.3%)	71 (1.9%)
Unusual abdominal pain	299 (4.7%)	76 (1.2%)	135 (4.9%)	35 (1.2%)	164 (4.5%)	41 (1.1%)
Diarrhoea	299 (4.7%)	85 (1.3%)	117 (4.2%)	38 (1.4%)	182 (5.0%)	47 (1.3%)
Headaches	1,858 (29.0%)	353 (5.4%)	789 (28.6%)	164 (5.8%)	1,069 (29.3%)	189 (5.1%)
Confusion, disorientation or drowsiness	476 (7.4%)	53 (0.8%)	164 (5.9%)	14 (0.5%)	312 (8.6%)	39 (1.1%)
Unusual eye-soreness	393 (6.1%)	54 (0.8%)	145 (5.3%)	24 (0.9%)	248 (6.8%)	30 (0.8%)
Skipping meals	782 (12.2%)	111 (1.7%)	279 (10.1%)	36 (1.3%)	503 (13.8%)	75 (2.0%)
Dizziness or light-headedness	1,023 (16.0%)	180 (2.8%)	376 (13.6%)	75 (2.7%)	647 (17.7%)	105 (2.8%)
Sore throat	1,418 (22.1%)	397 (6.1%)	589 (21.3%)	186 (6.6%)	829 (22.7%)	211 (5.7%)
Unusual strong muscle pains	670 (10.5%)	69 (1.1%)	232 (8.4%)	29 (1.0%)	438 (12.0%)	40 (1.1%)
Earache or ringing in ears	304 (4.7%)	66 (1.0%)	119 (4.3%)	26 (0.9%)	185 (5.1%)	40 (1.1%)
Raised welts on skin or swelling	71 (1.1%)	21 (0.3%)	30 (1.1%)	9 (0.3%)	41 (1.1%)	12 (0.3%)
Red/purple sores/blisters on feet	50 (0.8%)	14 (0.2%)	20 (0.7%)	7 (0.3%)	30 (0.8%)	7 (0.2%)
Other	209 (3.3%)	35 (0.5%)	107 (3.9%)	18 (0.6%)	102 (2.8%)	17 (0.5%)
**Previous physical health ***						
Very poor or poor	107 (1.7%)	148 (2.3%)	29 (1.1%)	53 (1.9%)	78 (2.1%)	95 (2.6%)
Okay	1,212 (18.9%)	1,428 (21.8%)	425 (15.4%)	497 (17.7%)	787 (21.6%)	931 (25.0%)
Good or very good	5,088 (79.4%)	4,966 (75.9%)	2,305 (83.4%)	2,264 (80.5%)	2,783 (76.3%)	2,702 (72.5%)
**Previous mental health***						
Very poor or poor	516 (8.1%)	667 (10.2%)	113 (4.1%)	182 (6.5%)	403 (11.1%)	485 (13.0%)

Okay	1,790 (27.9%)	1,968 (30.1%)	597 (21.6%)	671 (23.9%)	1,193 (32.7%)	1,297 (34.8%)
Good or very good	4,101 (64.0%)	3,907 (59.7%)	2,049 (74.3%)	1,961 (69.7%)	2,052 (56.3%)	1,946 (52.2%)
**Self-rated health****	90 (85, 100)	90 (80, 100)	95 (85, 100)	95 (85, 100)	90 (80,95)	90 (80, 95)

Note. Data are n (%). *Participants were asked "How was your physical/mental health in general before your Covid-19 test?" in two separate questions using a five-category Likert scale; we recoded these variables into three categories (very poor and poor, okay, and good and very good); questions were not asked in relation to 6 months post-test. ** reported as median(IQR), measured by a visual analogue scale (EQ-5D VAS score), which records responses to the question "Please look at the scale and select the number for your health BEFORE your Covid-19 test and your health TODAY" participants are told that "100% means the best health you can think of; 0% means the worst health you can think of"

Pinto Pereira SM, *et al. Arch Dis Child* 2023; 108:289–295. doi: 10.1136/archdischild-2022-324656

Supplement Table 3. Reported symptoms at 6 months by SARS-CoV-2 status, overall and stratified by age group

	All participants		Participants aged 11-14 years		Participants aged 15-17	
	**Tested positive for SARS-CoV-2 (n = 6,407)**	**Tested negative for SARS-CoV-2 (n = 6,542)**	**Tested positive for SARS-CoV-2 (n = 2,759)**	**Tested negative for SARS-CoV-2 (n = 2,814)**	**Tested positive for SARS-CoV-2 (n = 3,648)**	**Tested negative for SARS-CoV-2 (n = 3,728)**
No reported symptoms	2,507 (39.1%)	3,713 (56.8%)	1,253 (45.4%)	1,748 (62.1%)	1,254 (34.4%)	1,965 (52.7%)
1 symptom	1,355 (21.2%)	1,228 (18.8%)	562 (20.4%)	489 (17.4%)	793 (21.7%)	739 (19.8%)
2 symptoms	779 (12.2%)	563 (8.6%)	308 (11.7%)	206 (7.3%)	471 (12.9%)	357 (9.6%)
3 symptoms	569 (8.9%)	318 (4.9%)	224 (8.1%)	121 (4.3%)	345 (9.5%)	197 (5.3%)
4 symptoms	393 (6.1%)	216 (3.3%)	155 (5.6%)	78 (2.8%)	238 (6.5%)	138 (3.7%)
≥5 symptoms	804 (12.6%)	504 (7.7%)	257 (9.3%)	172 (6.1%)	547 (15.0%)	332 (8.9%)
**Specific symptoms**						
Fever	82 (1.3%)	96 (1.5%)	34 (1.2%)	28 (1.0%)	48 (1.3%)	68 (1.8%)
Chills	479 (7.5%)	387 (5.9%)	193 (7.0%)	164 (5.8%)	286 (7.8%)	223 (6.0%)
Persistent cough	215 (3.4%)	209 (3.2%)	91 (3.3%)	76 (2.7%)	124 (3.4%)	133 (3.6%)
Tiredness	2,458 (38.4%)	1,747 (26.7%)	866 (31.4%)	584 (20.8%)	1,592 (43.6%)	1,163 (31.2%)
Shortness of breath	1,462 (22.8%)	710 (10.9%)	462 (16.8%)	263 (9.4%)	1,000 (27.4%)	447 (12.0%)
Loss of smell	903 (14.1%)	90 (1.4%)	295 (10.7%)	34 (1.2%)	608 (16.7%)	56 (1.5%)
Unusually hoarse voice	98 (1.5%)	95 (1.5%)	45 (1.6%)	44 (1.6%)	53 (1.5%)	51 (1.4%)
Unusual chest pain	419 (6.5%)	234 (3.6%)	148 (5.4%)	95 (3.4%)	271 (7.4%)	139 (3.7%)
Unusual abdominal pain	288 (4.5%)	226 (3.5%)	98 (3.6%)	87 (3.1%)	190 (5.2%)	139 (3.7%)
Diarrhoea	181 (2.8%)	157 (2.4%)	55 (2.0%)	47 (1.7%)	126 (3.5%)	110 (3.0%)
Headaches	1,171 (18.3%)	825 (12.6%)	447 (16.2%)	311 (11.1%)	724 (19.9%)	514 (13.8%)
Confusion, disorientation or drowsiness	345 (5.4%)	218 (3.3%)	116 (4.2%)	75 (2.7%)	229 (6.3%)	143 (3.8%)
Unusual eye-soreness	356 (5.6%)	260 (4.0%)	124 (4.5%)	95 (3.4%)	232 (6.4%)	165 (4.4%)
Skipping meals	609 (9.5%)	448 (6.9%)	223 (8.1%)	145 (5.2%)	386 (10.6%)	303 (8.1%)
Dizziness or light-headedness	838 (13.1%)	535 (8.2%)	303 (11.0%)	189 (6.7%)	535 (14.7%)	346 (9.3%)
Sore throat	414 (6.5%)	406 (6.2%)	184 (6.7%)	164 (5.8%)	230 (6.3%)	242 (6.5%)
Unusual strong muscle pains	286 (4.5%)	163 (2.5%)	109 (4.0%)	72 (2.6%)	177 (4.9%)	91 (2.4%)
Earache or ringing in ears	407 (6.4%)	284 (4.3%)	167 (6.1%)	109 (3.9%)	240 (6.6%)	175 (4.7%)
Raised welts on skin or swelling	73 (1.1%)	47 (0.7%)	29 (1.1%)	17 (0.6%)	44 (1.2%)	30 (0.8%)
Red/purple sores/blisters on feet	66 (1.0%)	58 (0.9%)	25 (0.9%)	30 (1.1%)	41 (1.1%)	28 (0.8%)
Other	488 (7.6%)	384 (5.9%)	231 (8.4%)	172 (6.1%)	257 (7.0%)	212 (5.7%)
Self-rated health*	90 (80,95)	90 (80,100)	95 (80,100)	95 (85,100)	90 (75,95)	90 (75,95)

* reported as median(IQR), scored on a scale of 0 (worst) to 100 (best)

Pinto Pereira SM, *et al. Arch Dis Child* 2023; 108:289–295. doi: 10.1136/archdischild-2022-324656

Supplementary Table 4. Characteristics at baseline of CYP by SARS-CoV-2 status and whether meeting published Delphi consensus definition of long COVID* at 6 months

	**SARS-CoV-2 Test Negatives (N = 6,542)**		**SARS-CoV-2 Test Positives (N = 6,407)**	
	**Not meeting long COVID definition at 6months n (%)**	**Meeting long COVID definition at 6months n (%)**	**Not meeting long COVID definition at 6months n (%)**	**Meeting long COVID definition at 6months n (%)**
**All**	5,380 (82.2%)	1,162 (17.8%)	4,836 (75.5%)	1,571 (24.5%)
**Number of symptoms at testing**				
None	4,893 (82.6%)	1,028 (17.4%)	2,843 (76.1%)	893 (23.9%)
1 symptom	71 (92.2%)	6 (7.8%)	214 (89.5%)	25 (10.5%)
2 symptoms	86 (91.5%)	8 (8.5%)	231 (88.2%)	31 (11.8%)
3 symptoms	86 (87.8%)	12 (12.2%)	264 (88.9%)	33 (11.1%)
4 symptoms	69 (83.1%)	14 (16.9%)	261 (85.3%)	45 (14.7%)
≥5 symptoms	175 (65.1%)	94 (34.9%)	1,023 (65.3%)	544 (34.7%)
**Sex**				
Male	2,182 (89.8%)	248 (10.2%)	2,030 (84.1%)	385 (15.9%)
Female	3,198 (77.8%)	914 (22.2%)	2,806 (70.3%)	1,186 (29.7%)
**Age (years)**				
11-14	2,388 (84.9%)	426 (15.1%)	2,171 (78.7%)	588 (21.3%)
15-17	2,992 (80.3%)	736 (19.7%)	2,665 (73.1%)	983 (27.0%)
**Ethnicity**				
White	4,217 (83.0%)	866 (17.0%)	3,713 (75.5%)	1,208 (24.6%)
Asian or Asian British	690 (80.6%)	166 (19.4%)	708 (77.1%)	210 (22.9%)
Mixed	235 (77.1%)	70 (23.0%)	187 (70.6%)	78 (29.4%)
Black, African or Caribbean	138 (80.2%)	34 (19.8%)	119 (77.8%)	34 (22.2%)
Other	75 (80.7%)	18 (19.4%)	81 (72.3%)	31 (27.7%)
Prefer not to say	25 (75.8%)	8 (24.2%)	28 (73.7%)	10 (26.3%)
**Region**				
East Midlands	593 (83.5%)	117 (16.5%)	490 (76.2%)	153 (23.8%)
East of England	612 (82.5%)	130 (17.5%)	497 (76.6%)	152 (23.4%)
London	692 (84.0%)	132 (16.0%)	543 (74.9%)	182 (25.1%)
North East	294 (77.6%)	85 (22.4%)	293 (72.0%)	114 (28.0%)
North West	747 (81.2%)	173 (18.8%)	736 (75.0%)	245 (25.0%)
South East	749 (84.2%)	141 (15.8%)	672 (75.9%)	213 (24.1%)
South West	398 (81.4%)	91 (18.6%)	371 (74.5%)	127 (25.5%)
West Midlands	720 (82.1%)	157 (17.9%)	661 (78.0%)	186 (22.0%)
Yorkshire and the Humber	575 (80.9%)	136 (19.1%)	573 (74.2%)	199 (25.8%)
**IMD quintile**				

Pinto Pereira SM, *et al. Arch Dis Child* 2023; 108:289–295. doi: 10.1136/archdischild-2022-324656

1 (most deprived)	982 (76.4%)	304 (23.6%)	898 (70.8%)	370 (29.2%)
2	954 (81.2%)	221 (18.8%)	888 (76.0%)	281 (24.0%)
3	1,001 (82.1%)	219 (18.0%)	838 (74.8%)	282 (25.2%)
4	1,165 (85.0%)	205 (15.0%)	1,027 (76.6%)	313 (23.4%)
5 (least deprived)	1,278 (85.7%)	213 (14.3%)	1,185 (78.5%)	325 (21.5%)
**Previous Physical Health**				
Very poor or poor	89 (60.1%)	59 (39.9%)	58 (54.2%)	49 (45.8%)
Okay	1,011 (70.8%)	417 (29.2%)	763 (63.0%)	449 (37.1%)
Good or very good	4,280 (86.2%)	686 (13.8%)	4,015 (78.9%)	1,073 (21.1%)
**Previous mental health**				
Very poor or poor	349 (52.3%)	318 (47.7%)	241 (46.7%)	275 (53.3%)
Okay	1,521 (77.3%)	447 (22.7%)	1,188 (66.4%)	602 (33.6%)
Good or very good	3,510 (89.8%)	397 (10.2%)	3,407 (83.1%)	694 (16.9%)
**Self-rated Health****	95 (85,100)	80 (70,90)	95 (85,100)	90 (80,95)

*Using data from the questionnaire on symptoms and the EQ-5D-Y scale at the time of the questionnaire (i.e., approximately 6 months after the PCR-test), long COVID was operationalized as having at least 1 symptom and experiencing some/a lot of problems with respect to mobility, self-care, doing usual activities or having pain/discomfort or feeling very worried/sad. **reported as median(IQR), scored on a scale of 0 (worst) to 100 (best)

Pinto Pereira SM, *et al. Arch Dis Child* 2023; 108:289–295. doi: 10.1136/archdischild-2022-324656

Supplementary Table 5. Characteristics at 6 months post-test by SARS-CoV-2 status and and whether meeting published Delphi consensus definition of long COVID*

	SARS-CoV-2 Test Negatives (N=6,542)		SARS-CoV-2 Test Positives (N=6,407)	
	Not meeting long COVID definition at 6 months (n=5,380)	Meeting long COVID definition at 6 months (n=1,162)	Not meeting long COVID definition at 6 months (n=4,836)	Meeting long COVID definition at 6 months (n=1,571)
**SDQ**				
SDQ Total Difficulties				
Median	10	17	9	16
(25th, 75th)	(6, 14)	(13, 22)	(5, 13)	(11, 20)
SDQ Emotional symptoms				
Median	3	6	3	6
(25th, 75th)	(1, 5)	(4, 8)	(1, 4)	(4, 7)
SDQ Conduct problems				
Median	1	2	1	2
(25th, 75th)	(0, 2)	(1, 4)	(0, 2)	(1, 3)
SDQ Hyperactivity/inattention				
Median	4	6	3	5
(25th, 75th)	(2, 5)	(4, 8)	(2, 5)	(3, 7)
SDQ peer relationship problem				
Median	2	3	1	3
(25th, 75th)	(1, 3)	(2, 5)	(1, 3)	(1, 4)
**SWEMWBS**				
Median	21.5	18.0	22.4	19.3
(25th, 75th)	(19.3, 24.1)	(16.4, 20.7)	(19.3, 25.0)	(16.9, 21.5)
Mean (SD)	21.9 (4.2)	18.6 (3.6)	22.5 (4.2)	19.4 (3.7)
**Chalder fatigue scale**				
Median	11	17	11	17
(25th, 75th)	(11,14)	(14, 21)	(11, 14)	(14, 21)
Mean (SD)	11.9 (4.3)	17.7 (5.7)	12.0 (4.2)	17.7 (5.3)
**Self-rated health****	95 (85,100)	75 (60,85)	95 (85,100)	75 (65,85)

Note. SDQ = Strengths and Difficulties Questionnaire; SWEMBS = Short Warwick-Edinburgh Mental Wellbeing Scale.
*A higher SDQ score indicates more problems; a higher SWEMWBS score indicates better mental well-being; a higher fatigue score is more severe. Using data from the questionnaire on symptoms and the EQ-5D-Y scale at the time of the questionnaire (i.e., approximately 6 months after the PCR-test), long COVID was operationalized as having at least 1 symptom and experiencing some/a lot of problems with respect to mobility, self-care, doing usual activities or having pain/discomfort or feeling very worried/sad. Number of symptoms and the EQ-5D-Y scale at 6 months not shown in this table as they are part of the definition of long COVID. **reported as median(IQR), scored on a scale of 0 (worst) to 100 (best)

Pinto Pereira SM, *et al. Arch Dis Child* 2023; 108:289–295. doi: 10.1136/archdischild-2022-324656

Supplementary Table 6. Characteristics at baseline of CYP by baseline SARS-CoV-2 status and vaccination status by six months

	SARS-CoV-2 Test Negatives (N = 6,527)		SARS-CoV-2 Test Positives (N = 6,402)	
	Vaccine: No n (%)	Vaccine: Yes n (%)	Vaccine: No n (%)	Vaccine: Yes n (%)
**All**	5,832 (89.4%)	695 (10.7%)	5,826 (91.0%)	576 (9.0%)
**Number of symptoms**				
None	5,268 (89.2%)	638 (10.8%)	3,383 (90.7%)	348 (9.3%)
1 symptom	72 (93.5%)	5 (6.5%)	227 (95.0%)	12 (5.0%)
2 symptoms	86 (91.5%)	8 (8.5%)	253 (96.6%)	9 (3.4%)
3 symptoms	88 (89.8%)	10 (10.2%)	276 (92.9%)	21 (7.1%)
4 symptoms	77 (92.8%)	6 (7.2%)	284 (92.8%)	22 (7.2%)
≥5 symptoms	241 (89.6%)	28 (10.4%)	1,403 (89.5%)	164 (10.5)
**Sex**				
Male	2,216 (91.5%)	206 (8.5%)	2,257 (93.5%)	156 (6.5%)
Female	3,616 (88.1%)	489 (11.9%)	3,569 (89.5%)	420 (10.5%)
**Age (years)**				
11-14	2,775 (98.9%)	30 (1.1%)	2,745 (99.5%)	13 (0.5%)
15-17	3,057 (82.1%)	665 (17.9%)	3,081 (84.6%)	563 (15.5%)
**Ethnicity**				
White	4,487 (88.5%)	583 (11.5%)	4,467 (90.9%)	450 (9.2%)
Asian or Asian British	785 (91.9%)	69 (8.1%)	834 (91.0%)	83 (9.1%)
Mixed	277 (90.8%)	28 (9.2%)	243 (91.7%)	22 (8.3%)
Black, African or Caribbean	163 (94.8%)	9 (5.2%)	141 (92.2%)	12 (7.8%)
Other	89 (95.7%)	4 (4.3%)	104 (92.9%)	8 (7.1%)
Prefer not to say	31 (93.9%)	2 (6.1%)	37 (97.4%)	1 (2.6%)
**Region**				
East Midlands	632 (89.4%)	75 (10.6%)	581 (90.4%)	62 (9.6%)
East of England	644 (86.9%)	97 (13.1%)	585 (90.1%)	64 (9.9%)
London	738 (89.9%)	83 (10.1%)	657 (90.6%)	68 (9.4%)
North East	345 (91.0%)	34 (9.0%)	384 (94.4%)	23 (5.7%)
North West	817 (89.0%)	101 (11.0%)	890 (90.9%)	89 (9.1%)
South East	783 (88.3%)	104 (11.7%)	786 (89.1%)	96 (10.9%)
South West	423 (86.5%)	66 (13.5%)	447 (89.8%)	51 (10.2%)
West Midlands	798 (91.2%)	77 (8.8%)	784 (92.6%)	63 (7.4%)
Yorkshire and the Humber	652 (91.8%)	58 (8.2%)	712 (92.2%)	60 (7.8%)
**IMD quintile**				
1 (most deprived)	1,161 (90.5%)	122 (9.5%)	1,164 (91.9%)	103 (8.1%)
2	1,055 (89.8%)	120 (10.2%)	1,055 (90.4%)	112 (9.6%)
3	1,093 (89.9%)	123 (10.1%)	1,021 (91.2%)	98 (8.8%)

4	1,208 (88.4%)	158 (11.6%)	1,222 (91.3%)	117 (8.7%)
5 (least deprived)	1,315 (88.4%)	172 (11.6%)	1,364 (90.3%)	146 (9.7%)
**Previous physical health**				
Very poor or poor	125 (84.5%)	23 (15.5%)	90 (84.9%)	16 (15.1%)
Okay	1,225 (85.8%)	202 (14.2%)	1,080 (89.2%)	131 (10.8%)
Good or very good	4,482 (90.5%)	470 (9.5%)	4,656 (91.6%)	429 (8.4%)
**Previous mental health**				
Very poor or poor	561 (84.1%)	106 (15.9%)	451 (87.4%)	65 (12.6%)
Okay	1,720 (87.6%)	243 (12.4%)	1,585 (88.7%)	203 (11.4%)
Good or very good	3,551 (91.1%)	346 (8.9%)	3,790 (92.5%)	308 (7.5%)
**Self-rated health***	90 (80,100)	90 (80,95)	90 (85,100)	90 (80,95)

Note: Those who did not respond to the vaccine question (n=20) were excluded *reported as median(IQR), scored on a scale of 0 (worst) to 100 (best)

Pinto Pereira SM, *et al. Arch Dis Child* 2023; 108:289–295. doi: 10.1136/archdischild-2022-324656

Supplementary Table 7. Characteristics at 6 months post-test of CYP by baseline SARS-CoV-2 status and vaccination status by 6 months

	SARS-CoV-2 Test Negatives (N=6,527)		SARS-CoV-2 Test Positives (N=6,402)	
	Vaccine: No (n=5,832)	Vaccine: Yes (n=695)	Vaccine: No (n=5,826)	Vaccine: Yes (n=576)
**Number of symptoms**				
None	3,298 (56.6%)	405 (58.3%)	2,297 (39.4%)	208 (36.1%)
1 symptom	1,111 (19.1%)	113 (16.3%)	1,238 (21.3%)	115 (20.0%)
2 symptoms	504 (8.6%)	58 (8.4%)	694 (11.9%)	85 (14.8%)
3 symptoms	279 (4.8%)	39 (5.6%)	515 (8.8%)	54 (9.4%)
4 symptoms	200 (3.4%)	16 (2.3%)	360 (6.2%)	33 (5.7%)
≥5 symptoms	440 (7.5%)	64 (9.2%)	722 (12.4%)	81 (14.1%)
**Meeting long COVID definition***				
No	4,802 (82.3%)	564 (81.2%)	4,422 (75.9%)	410 (71.2%)
Yes	1,030 (17.7%)	131 (18.9%)	1,404 (24.1%)	166 (28.8%)
**EQ5DY**				
Some/lots of mobility problems	339 (5.8%)	56 (8.1%)	330 (5.7%)	50 (8.7%)
Some/lots of self-care problems	296 (5.1%)	47 (6.8%)	194 (3.3%)	22 (3.8%)
Some/lots of problems with usual activities	757 (13.0%)	101 (14.5%)	850 (14.6%)	93 (16.2%)
Some/lots of pain discomfort	860 (14.8%)	144 (20.7%)	972 (16.7%)	117 (20.3%)
A bit/very worried, sad/unhappy	2,351 (40.3%)	324 (46.6%)	2306 (39.6%)	46.4 (46.4%)
**SDQ**				
SDQ Total Difficulties				
Median	11	12	10	11
(25th, 75th)	(6, 16)	(8, 17)	(6, 15)	(7, 16)
SDQ Emotional symptoms				
Median	3	4	3	4
(25th, 75th)	(1, 5)	(2, 6)	(1, 5)	(2, 6)
SDQ Conduct problems				
Median	1	1	1	1
(25th, 75th)	(0, 2)	(0, 2)	(0, 2)	(0, 2)
SDQ Hyperactivity/inattention				
Median	4	4	4	4
(25th, 75th)	(2, 6)	(2, 6)	(2, 6)	(2, 6)
SDQ peer relationship problem				
Median	2	2	2	2
(25th, 75th)	(1, 3)	(1, 4)	(1, 3)	(1, 3)
**SWEMBS**				
Median	20.7	20.0	21.5	20.7
(25th, 75th)	(18.6, 24.1)	(18.0, 23.2)	(18.6, 24.1)	(18.6, 24.1)
Mean (SD)	21.4 (4.3)	20.7 (4.0)	21.8 (4.3)	21.2 (4.3)

Pinto Pereira SM, *et al. Arch Dis Child* 2023; 108:289–295. doi: 10.1136/archdischild-2022-324656

**Chalder fatigue scale**				
Median	11	12	12	13
(25th, 75th)	(11,15)	(11, 16)	(11, 16)	(11, 17)
Mean (SD)	12.9 (5.2)	13.5 (4.6)	13.3 (5.1)	14.2 (5.1)
**Self-rated health****	90 (80,100)	85 (75,95)	90 (80,95)	85 (75,95)

Note: Those who did not respond to the vaccine question (n=20) were excluded
* Using data from the questionnaire on symptoms and the EQ-5D-Y scale at the time of the questionnaire (i.e., approximately 6 months after the PCR-test), long COVID was operationalized as having at least 1 symptom and experiencing some/a lot of problems with respect to mobility, self-care, doing usual activities or having pain/discomfort or feeling very worried/sad. **reported as median(IQR), scored on a scale of 0 (worst) to 100 (best)

Pinto Pereira SM, *et al. Arch Dis Child* 2023; 108:289–295. doi: 10.1136/archdischild-2022-324656

Supplementary Table 8. Reweighted percentage (95% CIs)of reported symptom(s) at the time of test and at the 6 months questionnaire by SARS-CoV-2 status, overall and stratified by age-group.

	All participants (N=12,949)		Participants aged 11-14 (N=5,573)			Participants aged 15-17 (N=7,376)		
	**Reweighted SARS-CoV-2 Positive (n=6,407)**	**Reweighted SARS-CoV-2 Negative (n=6,542)**	**Reweighted all participants (n=5,573)**	**Reweighted SARS-CoV-2 Positive (n=2,759)**	**Reweighted SARS-CoV-2 Negative (n=2,814)**	**Reweighted all participants (n=7,376)**	**Reweighted SARS-CoV-2 Positive (n=3,648)**	**Reweighted SARS-CoV-2 Negative (n=3,728)**
**At time of test**								
**Number of symptoms**								
None	58.5% (58.0%, 58.9%)	90.4% (90.1%, 90.6%)	75.6% (75.3%, 76.0%)	59.4% (58.8%, 60.0%)	88.9% (88.5%, 89.2%)	76.3% (75.9%, 76.6%)	57.6% (57.0%, 58.2%)	91.8% (91.5%, 92.1%)
1 symptom	3.8% (3.6%, 4.0%)	1.3% (1.2%, 1.4%)	2.9% (2.8%, 3.0%)	3.9% (3.7%, 4.2%)	2.0% (1.9%, 2.2%)	2.0% (1.9%, 2.1%)	3.7% (3.5%, 3.9%)	0.6% (0.5%, 0.6%)
2 symptoms	4.2% (4.0%, 4.4%)	1.6% (1.5%, 1.7%)	3.3% (3.1%, 3.5%)	4.7% (4.5%, 5.0%)	2.1% (2.0%, 2.3%)	2.3% (2.1%, 2.4%)	3.7% (3.5%, 3.9%)	1.1% (0.9%, 1.2%)
3 symptoms	4.6% (4.4%, 4.8%)	1.5% (1.4%, 1.6%)	3.3% (3.2%, 3.5%)	5.0% (4.8%, 5.3%)	1.9% (1.7%, 2.1%)	2.6% (2.4%, 2.7%)	4.3% (4.0%, 4.5%)	1.2% (1.0%, 1.3%)
4 symptoms	4.9% (4.7%, 5.1%)	1.2% (1.1%,1.3%)	3.2% (3.1%, 3.4%)	5.5% (5.2%, 5.8%)	1.4% (1.3%, 1.6%)	2.5% (2.4%, 2.7%)	4.5% (4.2%, 4.7%)	0.9% (0.8%, 1.1%)
≥ 5 symptoms	24.0% (23.6%, 24.4%)	4.1% (3.9%, 4.2%)	11.6% (11.4%, 11.9%)	21.4% (20.9%, 21.9%)	3.7% (3.4%, 3.9%)	14.4% (14.1%, 14.7%)	26.3% (25.7%, 26.8%)	4.5% (4.3%, 4.7%)
**Specific symptoms**								
Fever	20.2% (19.9%, 20.6%)	4.3% (4.1%, 4.4%)	11.7% (11.4%, 11.9%)	20.6% (20.1%, 21.2%)	4.4% (4.1%, 4.6%)	11.3% (11.0%, 11.5%)	19.9% (19.4%, 20.4%)	4.2% (3.9%, 4.4%)
Chills	15.4% (15.1%, 15.8%)	2.5% (2.4%, 2.7%)	7.7% (7.5%, 7.8%)	14.6% (14.1%, 15.0%)	2.2% (2.0%, 2.4%)	8.9% (8.7%, 9.1%)	16.2% (15.7%, 16.6%)	2.9% (2.7%, 3.1%)
Persistent cough	15.4% (15.1%, 15.7%)	5.0% (4.8%, 5.1%)	9.0% (8.8%, 9.3%)	13.1% (12.6%, 13.5%)	5.7% (5.4%, 6.0%)	10.2% (10.0%, 10.5%)	17.4% (17.0%, 17.9%)	4.3% (4.1%, 4.5%)
Tiredness	24.1% (23.7%, 24.5%)	3.4% (3.3%, 3.6%)	11.6% (11.3%, 11.9%)	21.7% (21.2%, 22.3%)	3.3% (3.1%, 3.5%)	13.8 (13.5%, 14.1%)	26.3% (25.7%, 26.8%)	3.5% (3.3%, 3.7%)
Shortness of breath	11.5% (11.2%, 11.8%)	1.6% (1.5%, 1.7%)	4.1% (3.9%, 4.3%)	7.6% (7.2%, 7.9%)	1.3% (1.2%, 1.4%)	7.9% (7.6%, 8.1%)	15.0% (14.6%, 15.5%)	1.9% (1.8%, 2.1%)
Loss of smell	26.1% (25.7%, 26.5%)	1.9% (1.8%, 2.0%)	10.9% (10.6%, 11.1%)	21.9% (21.4%, 22.5%)	1.9% (1.7%, 2.1%)	14.5% (14.2%, 14.8%)	29.9% (29.3%, 30.5%)	1.8% (1.7%, 2.0%)
Unusually hoarse voice	4.5% (4.3%, 4.7%)	1.2% (1.1%, 1.2%)	2.5% (2.4%, 2.7%)	4.0% (3.8%, 4.3%)	1.3% (1.2%, 1.5%)	2.8% (2.6%, 2.9%)	4.9% (4.7%, 5.2%)	1.0% (0.9%, 1.1%)
Unusual chest pain	8.6% (8.4%, 8.9%)	1.7% (1.6%, 1.8%)	3.3% (3.1%, 3.4%)	5.4% (5.1%, 5.7%)	1.6% (1.5%, 1.8%)	6.3% (6.1%, 6.5%)	11.6% (11.2%, 12.0%)	1.9% (1.7%, 2.0%)
Unusual abdominal pain	4.6% (4.4%, 4.8%)	1.2% (1.1%, 1.3%)	2.8% (2.6%, 2.9%)	4.7% (4.4%, 5.0%)	1.2% (1.1%, 1.4%)	2.6% (2.5%, 2.8%)	4.5% (4.2%, 4.8%)	1.1% (1.0%, 1.2%)

Pinto Pereira SM, *et al. Arch Dis Child* 2023; 108:289–295. doi: 10.1136/archdischild-2022-324656

Diarrhoea	4.9% (4.7%, 5.1%)	1.4% (1.3%, 1.5%)	2.9% (2.8%, 3.1%)	4.7% (4.5%, 5.0%)	1.4% (1.3%, 1.6%)	3.0% (2.9%, 3.2%)	5.0% (4.8%, 5.3%)	1.4% (1.3%, 1.5%)
Headaches	28.8% (28.4%, 29.2%)	5.3% (5.1%, 5.5%)	15.8% (15.5%, 16.1%)	28.4% (27.8%, 29.0%)	5.6% (5.3%, 5.9%)	16.0% (15.7%, 16.3%)	29.2% (28.7%, 29.8%)	5.1% (4.8%, 5.3%)
Confusion, disorientation or drowsiness	7.1% (6.9%, 7.4%)	0.8% (0.7%, 0.9%)	2.8% (2.7%, 3.0%)	5.7% (5.4%, 6.0%)	0.5% (0.4%, 0.6%)	4.4% (4.2%, 4.6%)	8.4% (8.1%, 8.8%)	1.1% (1.0%, 1.2%)
Unusual eye-soreness	6.0% (5.7%, 6.2%)	0.9% (0.8%, 0.9%)	2.6% (2.5%, 2.8%)	4.8% (4.5%, 5.1%)	0.9% (0.8%, 1.0%)	3.6% (3.5%, 3.8%)	7.0% (6.7%, 7.3%)	0.8% (0.7%, 1.0%)
Skipping meals	11.9% (11.6%, 12.1%)	1.7% (1.6%, 1.8%)	5.2% (5.0%, 5.4%)	10.1% (9.8%, 10.5%)	1.1% (1.0%, 1.3%)	7.3% (7.1%, 7.5%)	13.4% (13.0%, 13.8%)	2.2% (2.0%, 2.4%)
Dizziness or light-headedness	15.2% (14.9%, 15.6%)	2.7% (2.5%, 2.8%)	7.3% (7.1%, 7.5%)	13.1% (12.7%, 13.6%)	2.5% (2.3%, 2.7%)	9.3% (9.0%, 9.5%)	17.1% (16.6%, 17.6%)	2.8% (2.6%, 3.0%)
Sore throat	22.0% (21.7%, 22.4%)	6.1% (5.9%, 6.3%)	13.0% (12.7%, 13.3%)	21.0% (20.5%, 21.5%)	6.5% (6.2%, 6.8%)	13.5% (13.2%, 13.8%)	23.0% (22.5%, 23.5%)	5.7% (5.4%, 5.9%)
Unusual strong muscle pains	10.0% (9.7%, 10.3%)	1.1% (1.0%, 1.1%)	4.2% (4.1%, 4.4%)	8.3% (7.9%, 8.6%)	0.9% (0.8%, 1.1%)	5.9% (5.7%, 6.1%)	11.6% (11.2%, 12.0%)	1.2% (1.0%, 1.3%)
Earache or ringing in ears	4.6% (4.4%, 4.7%)	1.0% (0.9%, 1.1%)	2.3% (2.2%, 2.4%)	4.1% (3.8%, 4.3%)	0.9% (0.8, 1.0%)	2.9% (2.7%, 3.0%)	5.0% (4.7%, 5.3%)	1.1% (1.0%, 1.2%)
Raised welts on skin or swelling	1.2% (1.1%, 1.3%)	0.4% (0.3%, 0.4%)	0.8% (0.7%, 0.8%)	1.2% (1.1%, 1.4%)	0.4% (0.3%, 0.5%)	0.7% (0.6%, 0.8%)	1.1% (1.0%, 1.2%)	0.3% (0.3%, 0.4%)
Red/purple sores/blisters on feet	0.8% (0.7%, 0.9%)	0.2% (0.1%, 0.2)	0.4% (0.4%, 0.5%)	0.8% (0.7%, 0.9%)	0.2% (0.1%, -.2%)	0.5% (0.4%, 0.5%)	0.8% (0.7%, 1.0%)	0.2% (0.1%, 0.2%)
Other	3.0% (2.9%, 3.2%)	0.6% (0.5%, 0.7%)	2.0% (1.9%, 2.1%)	3.6% (3.4%, 3.9%)	0.7% (0.6%, 0.8%)	1.4% (1.3%, 1.5%)	2.5% (2.3%, 2.7%)	0.5% (0.4%, 0.6%)
**At time of 6-month questionnaire**								
**Number of symptoms**								
None	41.1% (40.7%, 41.6%)	58.0% (57.6%, 58.4%)	55.0% (54.6%, 55.4%)	46.3% (45.7%, 46.9%)	62.1% (61.5%, 62.6%)	46.2% (45.8%, 46.6%)	36.5% (35.9%, 37.1%)	54.3% (53.8%, 54.8%)
1 symptom	20.8% (20.4%, 21.1%)	18.5% (18.2%, 18.8%)	18.4% (18.1%, 18.7%)	19.7% (19.2%, 20.2%)	17.4% (17.0%, 17.8%)	20.5% (20.2%, 20.8%)	21.8% (21.3%, 22.3%)	19.5% (19.0%, 19.9%)
2 symptoms	11.9% (11.6%, 12.2%)	8.3% (8.1%, 8.5%)	9.1% (8.9%, 9.4%)	11.3% (10.9%, 11.7%)	7.4% (7.1%, 7.8%)	10.7% (10.4%, 10.9%)	12.5% (12.1%, 12.9%)	9.2% (8.9%, 9.5%)
3 symptoms	8.5% (8.2%, 8.7%)	4.7% (4.5%, 4.8%)	5.9% (5.7%, 6.1%)	8.1% (7.8%, 8.5%)	4.2% (3.9%, 4.4%)	6.8% (6.6%, 7.0%)	8.7% (8.4%, 9.1%)	5.1% (4.9%, 5.4%)
4 symptoms	5.9% (5.7%, 6.1%)	3.3% (3.1%, 3.4%)	4.1% (4.0%, 4.3%)	5.5% (5.3%, 5.9%)	3.0% (2.8%, 3.2%)	4.7% (4.6%, 4.9%)	6.2% (5.9%, 6.5%)	3.5% (3.3%, 3.7%)
≥ 5 symptoms	11.8% (11.5%, 12.1%)	7.3% (7.1%, 7.5%)	7.4% (7.1%, 7.6%)	9.0% (8.7%, 9.4%)	6.0% (5.7%, 6.3%)	11.1% (10.8%, 11.3%)	14.3% (13.8%, 14.7%)	8.5% (8.2%, 8.8%)
**Specific symptoms**								
Fever	1.3%	1.5%	1.2%	1.3%	1.1%	1.7%	1.4%	2.0%

	(1.2%, 1.4%)	(1.4%, 1.6%)	(1.1%, 1.3%)	(1.1%, 1.4%)	(0.9%, 1.2%)	(1.6%, 1.8%)	(1.2%, 1.5%)	(1.8%, 2.1%)
Chills	7.1%<br>(6.9%, 7.3%)	5.9%<br>(5.7%, 6.1%)	6.3%<br>(6.1%, 6.5%)	6.9%<br>(6.5%, 7.2%)	5.9%<br>(5.6%, 6.2%)	6.6%<br>(6.3%, 6.8%)	7.3%<br>(7.0%, 7.6%)	5.9%<br>(5.7%, 6.2%)
Persistent cough	3.5%<br>(3.3%, 3.7%)	3.3%<br>(3.2%, 3.5%)	3.2%<br>(3.0%, 3.3%)	3.5%<br>(3.2%, 3.7%)	2.9%<br>(2.7%, 3.1%)	3.6%<br>(3.5%, 3.8%)	3.5%<br>(3.3%, 3.8%)	3.7%<br>(3.5%, 3.9%)
Tiredness	36.6%<br>(36.2%, 37.0%)	25.3%<br>(25.0%. 25.7%)	25.1%<br>(24.7%, 25.5%)	30.9%<br>(30.3%, 31.5%)	20.4%<br>(19.9%, 20.9%)	35.2%<br>(34.8%, 35.6%)	41.7%<br>(41.1%, 42.3%)	29.8%<br>(29.3%, 30.2%)
Shortness of breath	21.8%<br>(21.4%, 22.2%)	10.4%<br>(10.2%, 10.7%)	12.7%<br>(12.5%, 13.0%)	17.1%<br>(16.6%, 17.6%)	9.2%<br>(8.9%, 9.5%)	18.1%<br>(17.8%, 18.5%)	26.1%<br>(25.5%, 26.6%)	11.6%<br>(11.2%, 11.9%)
Loss of smell	13.1%<br>(12.8%, 13.4%)	1.4%<br>(1.3%, 1.5%)	5.2%<br>(5.0%, 5.4%)	10.0%<br>(9.6%, 10.4%)	1.2%<br>(1.1%, 1.4%)	8.1%<br>(7.8%, 8.3%)	15.8%<br>(15.4%, 16.3%)	1.6%<br>(1.5%, 1.8%)
Unusually hoarse voice	1.5%<br>(1.4%, 1.6%)	1.4%<br>(1.3%, 1.4%)	1.6%<br>(1.5%, 1.7%)	1.6%<br>(1.5%, 1.8%)	1.6%<br>(1.4%, 1.7%)	1.2%<br>(1.2%, 1.3%)	1.3%<br>(1.1%, 1.5%)	1.2%<br>(1.0%, 1.3%)
Unusual chest pain	6.0%<br>(5.8%, 6.2%)	3.3%<br>(3.2%, 3.5%)	3.9%<br>(3.8%, 4.1%)	4.9%<br>(4.6%, 5.2%)	3.2%<br>(3.0%, 3.4%)	5.1%<br>(4.9%, 5.3%)	7.0%<br>(6.7%, 7.3%)	3.5%<br>(3.3%, 3.7%)
Unusual abdominal pain	4.2%<br>(4.0%, 4.4%)	3.2%<br>(3.1%, 3.4%)	3.1%<br>(3.0%, 3.3%)	3.3%<br>(3.1%, 3.5%)	3.0%<br>(2.8%, 3.2%)	4.1%<br>(4.0%, 4.3%)	5.0%<br>(4.7%, 5.2%)	3.5%<br>(3.3%, 3.7%)
Diarrhoea	2.9%<br>(2.7%, 3.0%)	2.2%<br>(2.1%, 2.4%)	1.9%<br>(1.7%, 2.0%)	2.1%<br>(1.9%, 2.2%)	1.7%<br>(1.6%, 1.9%)	3.1%<br>(3.0%, 3.3%)	3.6%<br>(3.3%, 3.8%)	2.7%<br>(2.6%, 2.9%)
Headaches	17.5%<br>(17.2%, 17.9%)	12.1%<br>(11.8%, 12.4%)	13.3%<br>(13.0%, 13.6%)	15.9%<br>(15.5%, 16.4%)	11.2%<br>(10.9%, 11.6%)	15.6%<br>(15.3%, 15.9%)	18.9%<br>(18.5%, 19.4%)	12.9%<br>(12.5%, 13.3%)
Confusion, disorientation or drowsiness	5.2%<br>(5.0%, 5.4%)	3.1%<br>(3.0%, 3.2%)	3.3%<br>(3.2%, 3.5%)	4.2%<br>(4.0%, 4.5%)	2.6%<br>(2.4%, 2.8%)	4.7%<br>(4.5%, 4.9%)	6.0%<br>(5.7%, 6.3%)	3.6%<br>(3.4%, 3.8%)
Unusual eye-soreness	5.2%<br>(5.0%, 5.4%)	3.7%<br>(3.5%, 3.8%)	3.7%<br>(3.5%, 3.8%)	4.2%<br>(4.0%, 4.5%)	3.2%<br>(3.0%, 3.4%)	5.0%<br>(4.8%, 5.1%)	6.0%<br>(5.7%, 6.3%)	4.1%<br>(3.9%, 4.3%)
Skipping meals	9.1%<br>(8.9%, 9.4%)	6.4%<br>(6.2%, 6.6%)	6.4%<br>(6.2%, 6.6%)	8.0%<br>(7.6%, 8.3%)	5.2%<br>(4.9%, 5.4%)	8.7%<br>(8.5%, 9.0%)	10.2%<br>(9.8%, 10.5%)	7.5%<br>(7.2%, 7.8%)
Dizziness or light-headedness	11.9%<br>(11.6%, 12.2%)	7.6%<br>(7.4%, 7.8%)	8.2%<br>(7.9%, 8.4%)	10.2%<br>(9.8%, 10.6%)	6.5%<br>(6.3%, 6.8%)	10.7%<br>(10.5%, 11.0%)	13.5%<br>(13.0%, 13.9%)	8.5%<br>(8.2%, 8.8%)
Sore throat	6.4%<br>(6.2%, 6.6%)	6.1%<br>(5.9%, 6.3%)	6.3%<br>(6.1%, 6.6%)	6.8%<br>(6.5%, 7.1%)	6.0%<br>(5.7%, 6.2%)	6.2%<br>(6.0%, 6.4%)	6.0%<br>(5.8%, 6.3%)	6.3%<br>(6.0%, 6.5%)
Unusual strong muscle pains	4.3%<br>(4.2%, 4.5%)	2.5%<br>(2.3%, 2.6%)	3.1%<br>(3.0%, 3.3%)	3.9%<br>(3.6%, 4.1%)	2.5%<br>(2.3%, 2.7%)	3.5%<br>(3.3%, 3.6%)	4.8%<br>(4.5%, 5.0%)	2.4%<br>(2.3%, 2.6%)
Earache or ringing in ears	6.1%<br>(5.9%, 6.3%)	4.3%<br>(4.1%, 4.4%)	4.9%<br>(4.7%, 5.1%)	6.2%<br>(5.9%, 6.5%)	3.9%<br>(3.7%, 4.2%)	5.2%<br>(5.1%, 5.4%)	6.1%<br>(5.8%, 6.4%)	4.6%<br>(4.4%, 4.8%)
Raised welts on skin or swelling	1.1%<br>(1.0%, 1.2%)	0.7%<br>(0.6%, 0.7%)	0.8%<br>(0.7%, 0.8%)	1.1%<br>(1.0%, 1.2%)	0.5%<br>(0.4%, 0.6%)	0.9%<br>(0.9%, 1.0%)	1.1%<br>(1.0%, 1.3%)	0.8%<br>(0.8%, 0.9%)
Red/purple sores/blisters on feet	0.9%<br>(0.8%, 1.0%)	0.8%<br>(0.7%, 0.9%)	0.9%<br>(0.8%, 1.0%)	0.8%<br>(0.7%, 0.9%)	0.9%<br>(0.8%, 1.0%)	0.8%<br>(0.8%, 0.9%)	1.0%<br>(0.9%, 1.1%)	0.7%<br>(0.6%, 0.8%)
Other	7.4%<br>(7.2%, 7.7%)	5.9%<br>(5.7%, 6.1%)	7.2%<br>(6.9%, 7.4%)	8.0%<br>(7.7%, 8.4%)	6.4%<br>(6.2%, 6.7%)	6.1%<br>(5.9%, 6.3%)	6.9%<br>(6.6%, 7.2%)	5.4%<br>(5.2%, 5.7%)
